# Structure and Reactivity of Cu_2_O Nanocubes in Ethanol Dehydrogenation

**DOI:** 10.1021/acscatal.5c06573

**Published:** 2025-10-21

**Authors:** Van-Canh Nguyen, Eduardo Ortega, Daniel Cruz, Jie Zhu, Wiebke Frandsen, Shamil Shaikhutdinov, Beatriz Roldan Cuenya

**Affiliations:** Department of Interface Science, Fritz Haber Institute of the Max Planck Society, Faradayweg 4-6, 14195 Berlin, Germany

**Keywords:** Ethanol dehydrogenation, Copper catalysts, Cu nanocubes, Near ambient pressure X-ray photoelectron spectroscopy, Diffuse reflectance infrared Fourier transform spectroscopy

## Abstract

We investigated the structure and reactivity of cubic Cu_2_O nanoparticles (nanocubes) in the ethanol dehydrogenation (EDH) reaction, which is considered as an ecofriendly process for production of “green” hydrogen and valuable chemicals such as acetaldehyde. High-loaded catalysts prepared by physical mixing of nc-Cu_2_O and SiO_2_ demonstrated activity considerably higher than that of those prepared by conventional impregnation/calcination. Reactivity tests revealed the catalytic performance (conversion and selectivity) to be independent of the initial state of the catalyst, i.e., oxidized or reduced, due to the facile reduction of the Cu­(I) oxide to metallic Cu in the ethanol atmosphere, as observed by operando XRD, DRIFTS, and near ambient pressure (NAP) XPS. The reduction of nc-Cu_2_O is accompanied by strong morphological changes, i.e., the transformation of the nanocubes into roundish nanoparticles and their sintering as shown by TEM and SEM. The Cu­(I) oxide catalyst is initially active in EDH, but the Cu(0) phase formed in situ is considerably more active, and the Cu(0) phase is the only one that exists at the steady state. Analysis of the Cu surface by NAP XPS and CO DRIFTS revealed only the metallic state, with no indication for surface Cu oxide formation under the reaction conditions. The catalysts are stable at relatively low temperatures (∼170 °C) but deactivate, most notably at temperatures above 230 °C, due to coke (mostly amorphous carbon) formation. The results suggest that nc-Cu_2_O can be used as a well-defined precursor for the synthesis of high-loaded Cu catalysts for alcohol dehydrogenation at moderate temperatures.

## Graphical abstract

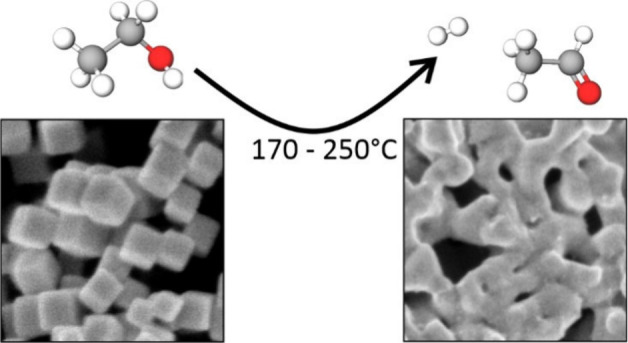

## 1 Introduction

Climate changes drive an intense search for alternative and sustainable energy sources, in particular of hydrogen. However, its storage, transportation, and safety issues prevent direct utilization of H_2_ in the gas phase. Instead, the use of hydrogen-containing liquid chemicals that can release hydrogen via catalytic dehydrogenation reactions is currently being considered as an alternative and more efficient approach.^1,2^ Among hydrogen carriers, ethanol seems to be well suited because it has a high hydrogen capacity and can be synthesized from renewable biomass.^3,4^ In addition to its low cost, ethanol has also the advantage of nontoxicity and easy storage. Therefore, the production of hydrogen from ethanol via steam reforming (C_2_H_5_OH + 3H_2_O → 2CO_2_ + 6H_2_) has received remarkable attention.^5^ However, this reaction occurs at high temperatures (above 500 °C) that lead not only to high energy costs but, more importantly, to the production of undesirable “greenhouse” CO_2_. Note also, that the most active catalysts for ethanol steam reforming are based on quite expensive noble metals. In this respect, the ethanol dehydrogenation (EDH) reaction at moderate temperatures on non-noble transition metal catalysts is a promising route to produce hydrogen and also valuable chemicals, such as acetaldehyde, in a more eco-friendly process for its production as compared to the currently used Wacker process.^6^ Commonly accepted mechanisms for nonoxidative ethanol dehydrogenation to acetaldehyde on metals include (1) ethanol dissociation to surface ethoxy species and hydrogen, (2) hydrogen abstraction in ethoxy to form acetaldehyde that desorbs, and (3) associative desorption of H_2_. Step (2) is considered to be the rate-limiting step.^7–10^ The oxide supports can also participate in the reaction by facilitating the O–H bond activation.^11,12^

Among different metal catalysts explored in the literature for this reaction, copper demonstrated good catalytic performance.^13–15^ In particular, Cu catalysts supported on SiO_2_ showed high selectivity to acetaldehyde,^16,17^ which was attributed to the rather weak acid–base properties of silica as compared to other frequently used supports such as Al_2_O_3_, MgO, and ZrO_2_. The latter are involved in the secondary reactions, even resulting in C_2+_ products.^18–20^ Due to the rich redox properties of Cu, various Cu species may, in principle, be present or coexist under the reaction conditions. Although it is generally accepted that Cu(0) metal sites are responsible for the H_2_ formation, there are still debates on whether Cu­(I,II) oxidic species are involved in ethanol transformations, for example, by lowering the activation barriers for the C–H and/or O–H bond cleavage.^16,21^ For example, the beneficial effect of using copper phyllosilicate as a precursor for high loaded Cu catalysts was rationalized in terms of the stabilization of a particular structure and the formation of partially charged Cu­(δ+) species as a result of the strong interaction with silica.^17^ Also, the presence of Cu­(I) at the Cu/ZrO_2_ interface was invoked to explain enhanced selectivity toward ethyl acetate.^11,12^ In this respect, the controlled synthesis of Cu catalysts, resulting in dual Cu(0) and Cu­(I) sites, could be a promising strategy to improve the catalytic performance, as has recently been demonstrated for methanol steam reforming.^22^ Another interesting, yet unexplored aspect is whether the EDH reaction on Cu is sensitive to the particular surface structure,^7,23^ which implies synthesis and reactivity tests on Cu nanoparticles of different shape. To shed more light on these issues, here we employed Cu_2_O nanoparticulate catalysts with cubic shape (nanocubes, NCs) which expose well-defined (100) facets (Figure 1a). Catalysts based on Cu_2_O NCs have recently been shown promising for a number of reactions such as CO_2_ hydrogenation to methanol,^24^ CO_2_ electroreduction,^25^ propylene oxidation,^26^ and the water-gas shift reaction,^27^ to name a few.

**Figure 1:**
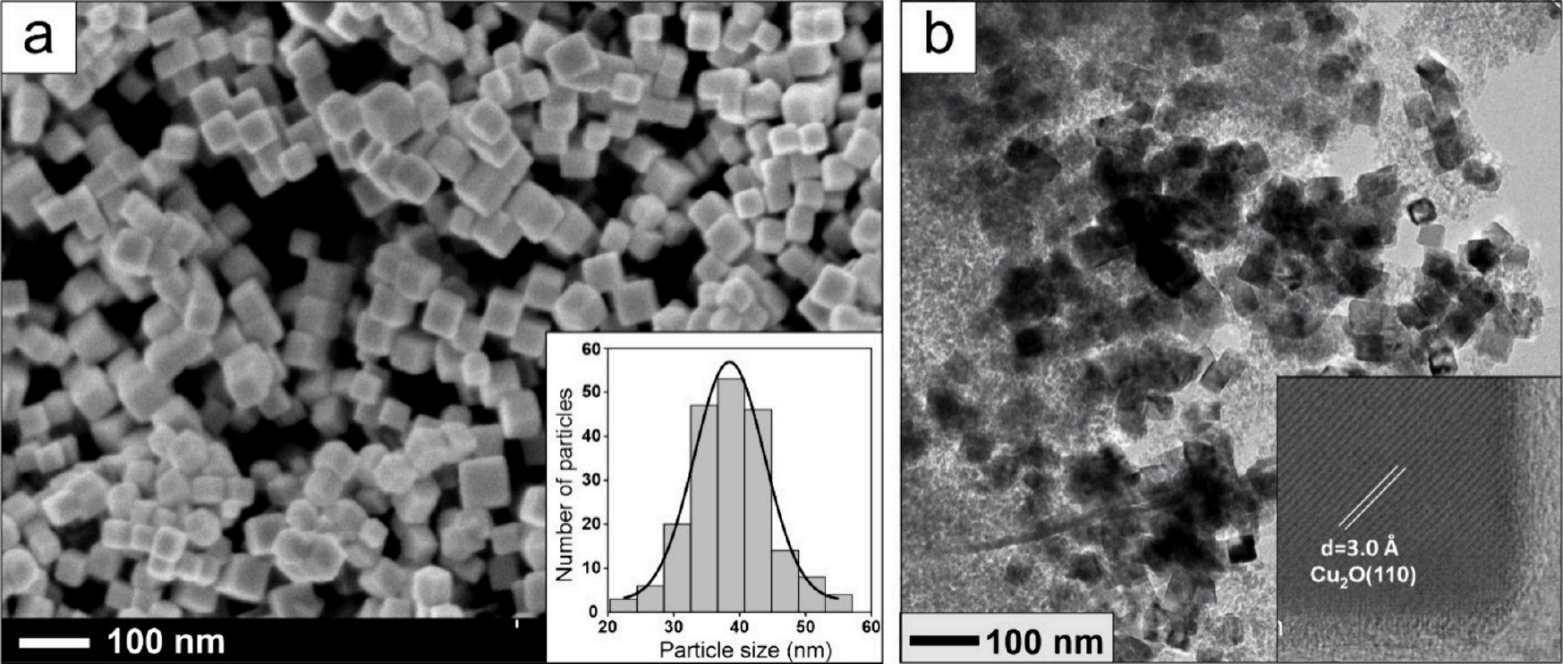
(a) SEM image and particle size distribution (inset) of the synthesized Cu_2_O nanocubes. (b) TEM image of the “as prepared” nc-Cu_2_O/SiO_2_ catalyst. A high-resolution image is shown in the inset.

In this work, we investigated the catalytic performance of nc-Cu_2_O catalysts in ethanol dehydrogenation at moderate temperatures (170–250 °C) to produce hydrogen and acetaldehyde. The structural and chemical evolution of the catalysts was investigated by operando X-ray diffraction (XRD), X-ray photoelectron spectroscopy (XPS), diffuse reflectance infrared Fourier transform spectroscopy (DRIFTS), and electron microscopy (TEM, SEM). The results show that ethanol acts as a strong reducing agent that transforms Cu­(I) oxide into metallic Cu(0) in the initial stage of the reaction. The reduction is accompanied by substantial morphological changes, such as the transformation of the nanocubes into roundish nanoparticles and their coalescence. Physical mixing of nc-Cu_2_O and nanocrystalline SiO_2_ diminishes, albeit cannot fully suppress, Cu sintering which is accompanied by Cu redispersion. Nonetheless, the nc-Cu_2_O based catalysts demonstrated intrinsic activity considerably higher than that of those prepared by conventional impregnation/calcination. The results suggest that Cu-oxide nanocubes are efficient well-defined precursors for the synthesis of high-loaded Cu catalysts.

## 2 Methods and Materials

The Cu_2_O nanocubes were synthesized as described elsewhere.^28^ More specifically, 30 mL of 0.2 M NaOH (Alfa Aesar) was mixed with 400 mL of deionized water, and then 10 mL of 0.1 M CuCl_2_ (Sigma-Aldrich, 99.95%) was added. Under constant stirring for 5 min, 20 mL of 0.1 M l-ascorbic acid (Sigma-Aldrich, 99%) was added, and the solution was continuously mixed for 1 h. The yellow solid was collected by centrifuge, and carefully washed by rinsing with deionized water and ethanol (99.9%). XPS spectra confirmed the absence of contamination (Na and Cl) in our samples (see Figure S1 in the Supporting Information). Nanocrystalline silica (Davisil grade 62, Sigma-Aldrich) was used for physical mixing with Cu_2_O NCs to prepare 30 wt % Cu_2_O/SiO_2_ catalyst, henceforth denoted as nc-Cu_2_O/SiO_2_.

The reference 30 wt % Cu/SiO_2_ catalyst was synthesized as follows. 4.5 g of Cu­(NO_3_)_2_·3H_2_O (Carl Roth) was dissolved in 100 mL of deionized water. Then 2.5 g of SiO_2_ (Davisil grade 62, Sigma-Aldrich) was added. Water was vaporized in a round flask connected to a rotary evaporator operated at 100 mbar and 50 °C. The obtained powder was calcined at 400 °C in synthetic air flow (100 mL/min) for 6 h.

The catalysts were tested in a fixed-bed flow reactor. For each test, 0.1 g of a catalyst was loaded into a tubular quartz reactor (6 mm inner diameter). Temperature was controlled by a PID controller (Eurotherm) using a thermocouple connected to the outer side of the quartz tube. Ethanol vapor was produced by a CEM evaporator (Bronkhorst) controlled by a liquid mass flow controller (mini CORI-FLOW, Bronkhorst) and mixed with N_2_. The catalyst was tested in a 200 mL/min N_2_ flow containing 0.5 g/h of ethanol. The gas composition was analyzed by online gas chromatography (GC, Agilent 6890A) equipped with TCD/FID detectors and PLOT-Q and Molecular Sieve 5A columns. To avoid condensation of ethanol and reaction products in the gas lines, the latter were kept at ∼100 °C using the heating tapes.

The conversion of ethanol (X, in %) was determined using the equation

$$X=\left(1-\frac{F_{EtOH}}{F_{EtOHref}}\right)\times 100=\left(1-\frac{A_{EtOH}\times F_{N_{2}}}{F_{EtOHref}\times f_{EtOH/N_{2}}}\right)\times 100$$

where *F_EtOH_* (mL/min) is the flow rate of ethanol, *A_EtOH_* is the integrated area of the ethanol peak area over nitrogen in the gas chromatogram, *F*_*N*_2__ (mL/min) is the flow rate of nitrogen, and *F_EtOHref_* (mL/min) and *f*_*EtOH/N*_2__ are respectively the flow rates of ethanol (calculated at room temperature via *PV = nRT*) and the calibration factor for ethanol, taking the *A*_*EtOH*_ signal at X = 0 as a reference.

The selectivity of acetaldehyde (*S*, in %) was determined using the equation

$$S=\left(\frac{F_{Ac}}{F_{EtOHref}-F_{EtOH}}\right)\times 100=\left(\frac{A_{Ac}\times F_{N_{2}}}{f_{Ac/N_{2}}}\right)\times \left(\frac{1}{F_{EtOHref}-F_{EtOH}}\right)\times 100$$

where *F_Ac_* (mL/min) is the flow rate of acetaldehyde at the effluent, *A_Ac_* is the integrated area of the acetaldehyde peak over nitrogen, and *f*_*Ac/N*_2__ is the calibration factor determined for acetaldehyde, taking the *A*_*Ac*_ signal at *X* = 100% and *S*_*Ac*_ = 100% as reference.

The production rate of acetaldehyde (*r*_*Ac*_) was determined as

$$r_{Ac}\;\left(g\;g_{cat}^{-1}\;h^{-1}\right)=\frac{F_{Ac}M_{Ac}}{V_{m}m_{cat}60}$$

where *M_Ac_* (=44 g mol^–1^) is the molecular mass of acetaldehyde, *V_m_* (=22.4 L mol^–1^) is molar volume of an ideal gas at STP, and *m_cat_* (g) is the catalyst’s weight.

The production rate of hydrogen (*r*_H_2__) was determined using the equation

$$r_{H_{2}}\left(\mathrm{mL}\;g_{cat}^{-1}\;min^{-1}\right)=\frac{A_{H_{2}}\times V_{N_{2}}}{f_{H_{2}/N_{2}}\times m_{cat}}$$ 4

where *A*_*H*_2__ is the integrated area of hydrogen over nitrogen in the gas chromatogram and *f*_H_2_/N_2__ is the calibration factor determined for hydrogen, taking the *A*_*H*_2__ signal assuming *X* = 100% and *S* = 100% as reference.

The turnover frequency of acetaldehyde and hydrogen (compound i) were determined using the equation

$$TOF=\frac{n_{i}\;\left(\mathrm{molecules}\;g_{cat}^{-1}\;s^{-1}\right)}{n_{Cu,SA}\;\left(\mathrm{atom}\;g_{cat}^{-1}\right)}=\frac{n_{i}\;\left(\mathrm{molecules}\;g_{cat}^{-1}\;s^{-1}\right)}{SA_{Cu}\;\left(m^{2}\;g_{cat}^{-1}\right)\;1.47\times 10^{19}\;\left(atom\;m^{-2}\right)}$$ 5

where *n_i_* is the molar production rate of compound *i*, *SA*_*Cu*_ is the copper surface area, and *n_Cu,SA_* is the average number of Cu surface atoms (1.5 × 10^19^ atom/m^2^) for principal low-index surfaces Cu(100), Cu(111), and Cu(110).

The Cu content in the catalyst was determined by inductively coupled plasma-mass spectrometry (ICP-MS). The sample (3–5 mg) was dissolved in 10 mL of H_2_SO_4_:HNO_3_:HCl (1:1:3 volume ratio). The solution was digested in a microwave (Anton Paar GmbH, Multiwave GO) at 180 °C for 30 min. The sample was filtered to remove solid particles and was finally diluted with water.

Specific Cu surface area in the reduced Cu/SiO_2_ catalysts was measured using N_2_O reactive frontal chromatography (2Cu + N_2_O → Cu_2_O + N_2_). The catalyst sample (0.2 g) was loaded into a stainless-steel reactor and reduced in situ in 10 vol % H_2_/He (50 mL/min) at 250 °C for 2 h and cooled to 30 °C in He flow. Then, 1 vol % N_2_O/He flow (5 mL/min) was introduced, and the gas composition was recorded by a quadrupole mass spectrometer (QMS, from Hiden). The surface area was calculated from the time delay of N_2_O (*m*/*e* = 44) compared to that of N_2_ (*m*/*e* = 28).

SEM images were obtained on a Hitachi S3400N microscope operated at 15 kV. High angle annular dark field (HAADF) STEM images were obtained with an FEI/TFS Titan microscope. The sample, taken from the catalytic reactor after different treatments, was physically dispersed onto a TEM grid that was put into the sample assembly, all in an inert atmosphere. The latter was opened inside the microscope.

XRD patterns were recorded on a Bruker AXS D8 Advance diffractometer equipped with a reaction cell (XRK 900, Anton Paar) using a Cu Kα source (λ = 1.5406 Å) and a position-sensitive energy-dispersive detector (LynxEye XE-T). The patterns were collected in the range of 2θ = 10–80° with an increment of 0.05°; the acquisition time was about 6 min. In *operando* XRD measurements, the reduction was performed in 10 vol % H_2_/N_2_ (50 mL/min). The EtOH/N_2_ vapor was produced by bubbling N_2_ (50 mL/min) through liquid ethanol kept at room temperature. The spectra were recorded in situ at stepwisely increased temperatures (Δ*T* = 20 °C; heating rate 5 °C/min). The crystalline particle size was determined by integral breadth method (LVol-IB) within Rietveld refinement as implemented in commercially available software (TOPAS, ver. 6).

XPS measurements were carried out in a UHV chamber (base pressure 5 × 10^–10^ mbar) using a monochromatic Al Kα (*h*ν = 1486.6 eV) X-ray source and a hemispherical analyzer (Phoibos 150). Cu_2_O nanocubes were drop-casted from a suspension in 2-propanol onto a Si(100) substrate (Sigert Wafer GmbH) covered by the native oxide layer. For quasi in situ XPS measurements, the samples were treated under catalytically relevant pressure and temperature conditions in a high-pressure cell (HPC 20, from Specs) attached to the analytical chamber through the gate valve. Synchrotron NAP-XPS experiments were performed at the ISSIS/UE48-EMIL beamline at BESSY (Berlin) using nc-Cu_2_O samples drop-casted onto a gold foil. The sample could be heated by an infrared laser from the backside. In both XPS setups, a chromel–alumel thermocouple spot welled to a stainless steel sample holder was used to measure the sample temperature. Hydrogen and ethanol were introduced using mass flow controllers (from Bronkhorst). The binding energies (BE) of the core levels were calibrated using the Fermi edge.

DRIFTS experiments were performed using a Bruker INVENIO R spectrometer equipped with a Harrick reaction cell (Praying Mantis CHC–CHA-5). A MCT detector was used to obtain spectra at 600–4000 cm^–1^ with an accumulation of 64 scans and a resolution of 2 cm^–1^. For DRIFTS of CO as a probe molecule, the cell was evacuated to ∼10^–6^ mbar, and then the sample was cooled to ∼−140 °C. The spectra were recorded at CO pressure increased from 0.1 to 10 mbar.

Raman spectra were measured with a confocal spectrometer (Renishaw, Virsa Analyzer) and a high-temperature Harrick cell (HVC-MRA-5). The spectra using the 785 nm laser excitation were recorded at an exposure time of 10 s, a laser power of 10 mW, and 3 accumulations. The spectrometer was calibrated on a Si wafer by setting the principal peak to 520.5 cm^–1^.

## 3 Results and Discussion

Figure 1a displays a SEM image of the synthesized Cu_2_O NCs exhibiting a narrow size distribution, with the edge length of 38.5 (±6) nm, on average. The particle morphology remains intact upon its mixing with a nanocrystalline SiO_2_ powder (Figure 1b).

The “as prepared” nc-Cu_2_O/SiO_2_ catalyst was tested in the ethanol dehydrogenation reaction at temperatures increased stepwise from 170 to 230 °C (Figure 2). The reactivity tests revealed hydrogen and acetaldehyde as the main products. The ethanol conversion and the production of H_2_ and acetaldehyde linearly increase with increasing temperature in this temperature range. For comparison, we also tested the catalysts pretreated in situ in 10 vol % H_2_/N_2_ at 170 and 250 °C prior to the reaction test. The temperatures were chosen on the basis of the temperature-programmed reduction profiles (Figure S2), suggesting partial Cu reduction at 170 °C and full reduction at 250 °C. The results revealed the catalytic performance to be almost independent of the degree of reduction of the initial catalysts. Nonetheless, the catalyst prereduced at 250 °C immediately showed steady-state activity, whereas the “as prepared” catalyst reached steady state after ca. 2 h at 170 °C (insets in Figure 2a,b). Since such kinetics was observed for the catalyst which was prereduced at 170 °C, it cannot be explained by pure thermal effect. In fact, these results suggest the “induction period” to be accompanied by the reaction-induced reduction of Cu_2_O to metallic Cu as the most active phase. To investigate the structural and chemical evolution of the Cu_2_O NCs in the reaction, we carried out XRD, TEM, XPS, and DRIFTS studies.

**Figure 2:**
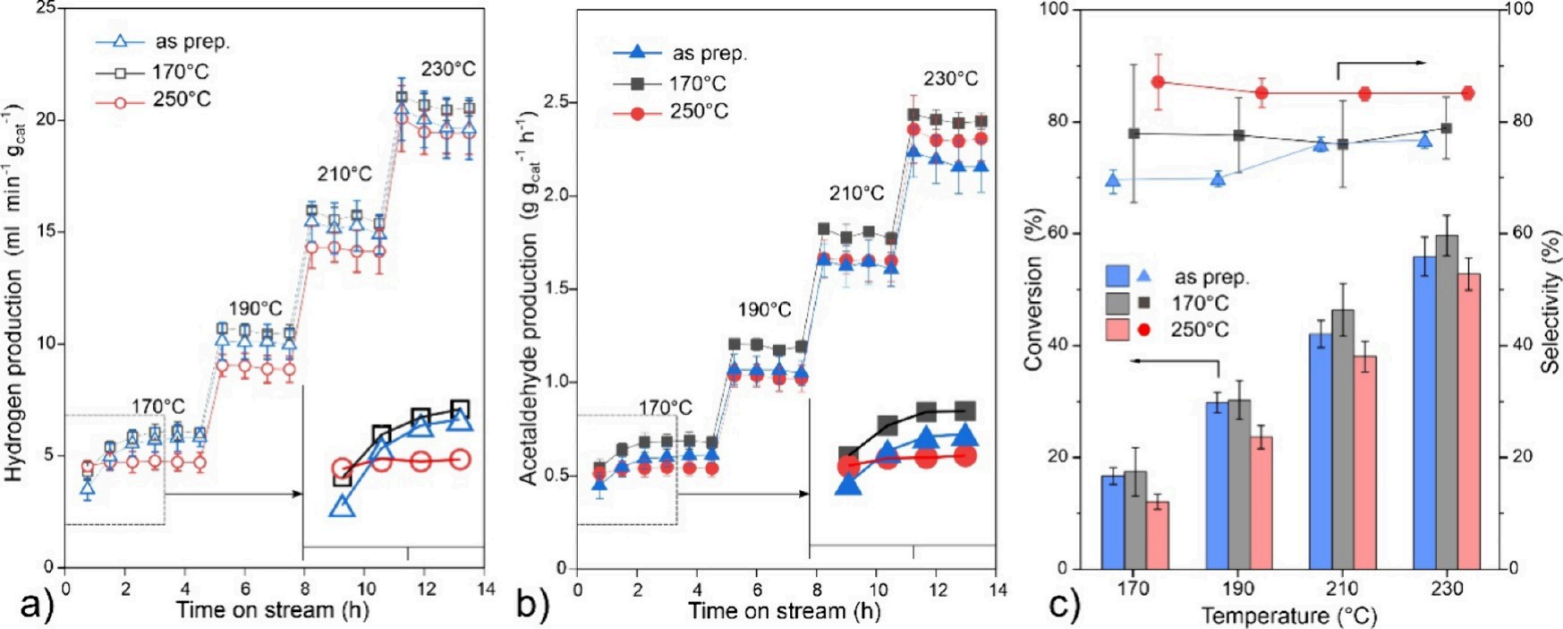
Reactivity of 30 wt % nc-Cu_2_O/SiO_2_ catalysts in ethanol dehydrogenation. The results are shown for the “as prepared” catalyst and the catalysts prereduced in situ in H_2_/N_2_ at 170 and 250 °C, respectively. (a,b) Kinetics of hydrogen and acetaldehyde production were measured at increasing reaction temperatures, from 170 to 230 °C. The initial period is zoomed in the insets. (c) Ethanol conversion and acetaldehyde selectivity measured at each temperature tested. In all panels, the error bars are obtained on the basis of several experiments.

### 3.1 X-ray Diffraction (XRD) and Transmission Electron Microscopy (TEM)

XRD pattern of the “as prepared” nc-Cu_2_O/SiO_2_ samples only showed diffraction peaks of Cu_2_O (see bottom spectra in Figure 3a,b). Using the widely used Scherrer equation and assuming the Scherrer constant (the shape factor) equal to 0.9, the average particle size of Cu_2_O nanocubes was found to be around 30 nm, in fair agreement with the microscopy data (Figure 1). In situ XRD patterns recorded during heating of the nc-Cu_2_O/SiO_2_ catalyst to 250 °C in the H_2_/N_2_ atmosphere are shown in Figure 3a. Up to 170 °C, the Cu_2_O phase is only observed in the spectra. At 190 °C, the Cu_2_O related peaks start to attenuate, while additional peaks corresponding to the metallic Cu phase appear and gain in intensity, so that only the metallic Cu phase is present at 250 °C (Table S1 in the Supporting Information). Note that the degree of reduction at intermediate temperatures depends on time. For example, the spectrum obtained for another sample treated at 170 °C for 60 min (dashed line in Figure 3a) looks very similar to that recorded immediately after reaching 190 °C.

**Figure 3:**
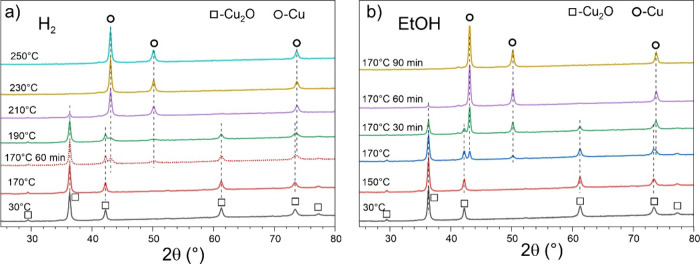
In situ XRD patterns obtained on 30 wt % nc-Cu_2_O/SiO_2_ catalyst in 10 vol % H_2_/N_2_ (a) and in EtOH/N_2_ (b) at temperatures increased stepwise. Panel (a) also shows the spectrum obtained on another sample heated in H_2_ at 170 °C for 60 min. In panel (b), the spectra at 170 °C were recorded at different reaction times, as indicated. The diffraction peaks corresponding to the Cu_2_O and Cu crystal phases are marked by squares and circles, respectively.

When exposed to ethanol vapor, the Cu_2_O phase was found to be stable up to 150 °C (Figure 3b). Upon heating to higher temperatures, the formation of metallic Cu sets in. Remarkably, the reduction in ethanol occurs at a considerably lower temperature than in H_2_ (170 °C vs 190 °C), indicating that ethanol behaves as a stronger reducing agent than H_2_. Moreover, the reduction at 170 °C develops over time, and it is almost completed after ca. 60 min, i.e., within the same time scale as the induction period observed in the reactivity tests (Figure 2a,b). Therefore, the induction period can be explained by the ethanol-induced reduction of the initial Cu_2_O catalyst into a fully metallic Cu phase, which is the only bulk phase that exists under the reaction conditions.

The results of TEM studies of the reduced and spent catalysts are summarized in Figure 4. Reduction in H_2_ causes substantial changes in the particle morphology, making them roundish. The particles coalescence depending on the homogeneity of the Cu_2_O and SiO_2_ physical mixture. In addition to the larger particles, we also observed particles much smaller than the original Cu_2_O nanocubes (some are marked in Figure 4). Such a redispersion (or fragmentation) of the Cu phase was also observed on the “as prepared” catalysts upon reaction with ethanol vapor at 170 °C. Apparently, the fragmentation occurs during the reduction process (either in H_2_ or ethanol) and likely involves Cu metal migration over the SiO_2_ support within the Ostwald ripening process. Overall, TEM images revealed substantial morphological changes (reshaping, coalescence, and redispersion) of the Cu_2_O NCs upon both reduction in H_2_ and reaction with ethanol.

**Figure 4:**
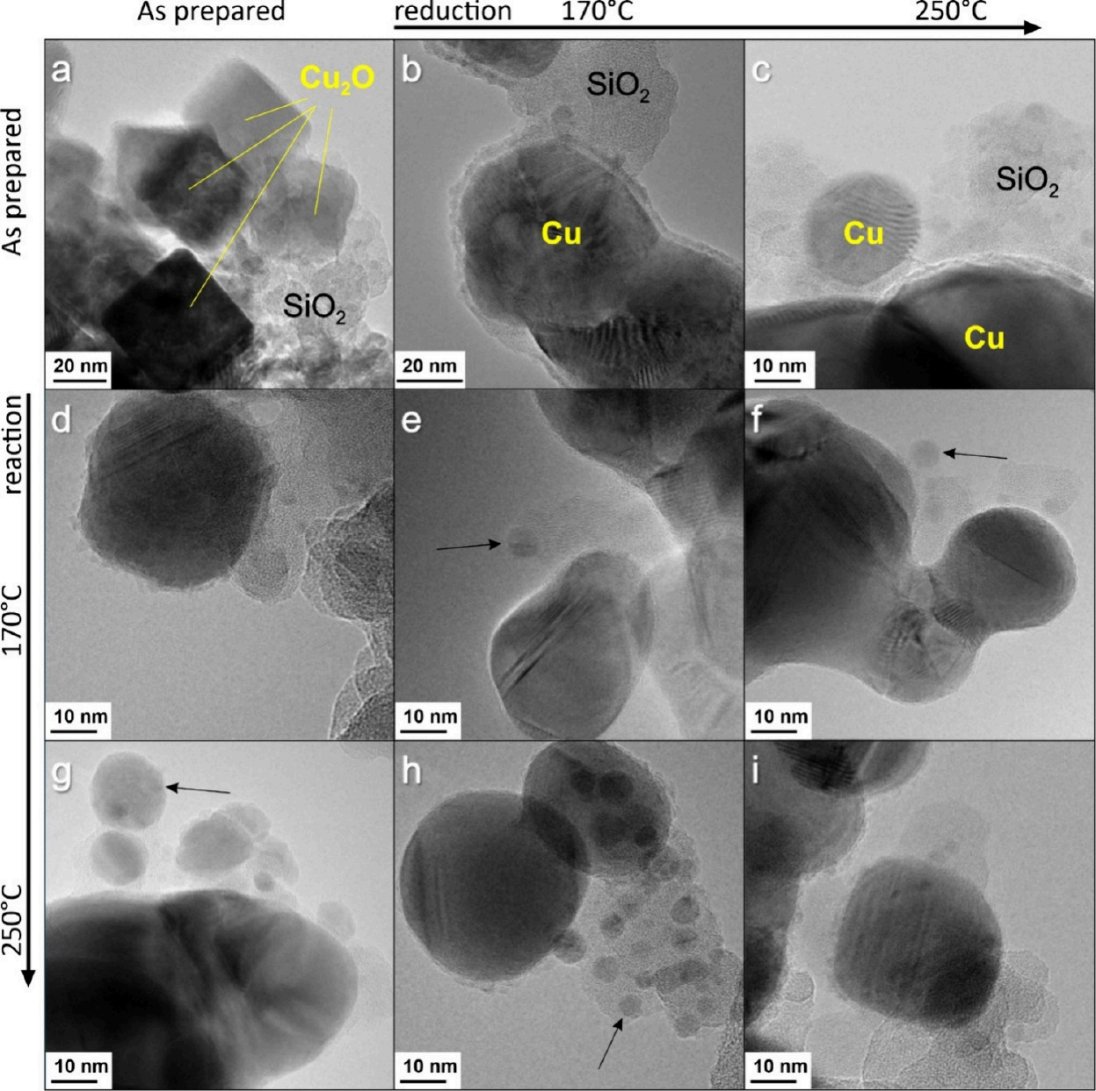
TEM images of nc-Cu_2_O/SiO_2_ catalysts: (a) as prepared, (b) after reduction at 170 °C, and (c) 250 °C for 2 h each. Images (d,g) show the “as prepared “ catalysts after reaction at 170°C (d) and 250°C (g), respectively. Images (e,h) show the catalysts prereduced at 170 °C after reaction at 170 °C (e) and 250 °C (h), respectively. Images (f,i) show the catalysts prereduced at 250 °C after reaction at 170 °C (f) and 250 °C (i), respectively. The reaction time was 12 h for all catalysts studied. During the reduction or reaction, the Cu particles are formed (some are marked by the arrows), which are considerably smaller than the original Cu_2_O nanocubes.

Table 1 compares the average domain size of the crystalline phases in nc-Cu_2_O/SiO_2_ catalysts (as prepared or prereduced in H_2_) before and after 12 h of the EDH reaction, which was determined by the integral breadth method (LVol-IB) within Rietveld refinement of XRD patterns (see Methods and Materials). The comparison revealed minor changes for a catalyst fully reduced at 250 °C prior to the reaction, whereas the crystallite size increased considerably for “as prepared” and mildly reduced (at 170 °C) catalysts. Tentatively, we assigned this effect to a higher reducing ability of ethanol that promotes Cu particle sintering and coalescence.

**Table 1:** Crystallite Size (in nm) of the Cu Phases in the 30 wt.% nc-Cu_2_O/SiO_2_ Catalysts Measured before and after the EDH Reaction Performed in the XRD Cell at 170°C and 250°C, Respectively, for 12 h^a^

Sample	as prepared	reduced at 170 °C	reduced at 250 °C
before reaction	21 (Cu_2_O)	21 (Cu_2_O) and 9 (Cu)	20 (Cu)
reaction at 170 °C	29 (Cu)	32 (Cu)	21 (Cu)
reaction at 250 °C	31 (Cu)	35 (Cu)	21 (Cu)

a The catalyst samples were used either “as prepared” or reduced in situ in H_2_ at 170 °C or 250 °C prior to the reaction test. For each experiment, fresh samples were used.

### 3.2 X-ray Photoelectron Spectroscopy (XPS)

The elemental composition and electronic state of the nc-Cu_2_O surface during various treatments was investigated by XPS using samples prepared by drop casting of Cu_2_O NCs onto a planar substrate, such as SiO_2_/Si­(001) and gold foil, from a suspension in 2-propanol. Survey spectra of the original samples revealed no impurities such as Na and Cl, but adventitious carbon (Figure S1, Figure S3a). The corresponding Cu 2p and Cu LMM Auger spectra (Figure 5a) showed the Cu­(I) state with a very small contribution of Cu­(II), most likely Cu­(OH)_2_ (see also the DRIFTS results below). Quasi in situ XPS spectra recorded after sample treatment in 1 bar of H_2_ at 170 °C for 30 min revealed both Cu­(I) and Cu(0) (44% and 56%, respectively), suggesting partial reduction of the surface, while the treatment at 250 °C led to the complete reduction, in full agreement with XRD data (Figure 3a). To monitor the surface state of nc-Cu_2_O in the reaction atmosphere, we employed synchrotron NAP-XPS. With the synchrotron source, it was also possible to measure soft X-ray absorption near-edge structure (XANES) spectra of the Cu L_3_ and L_2_ edges using a total electron yield (TEY) mode.^29^

**Figure 5:**
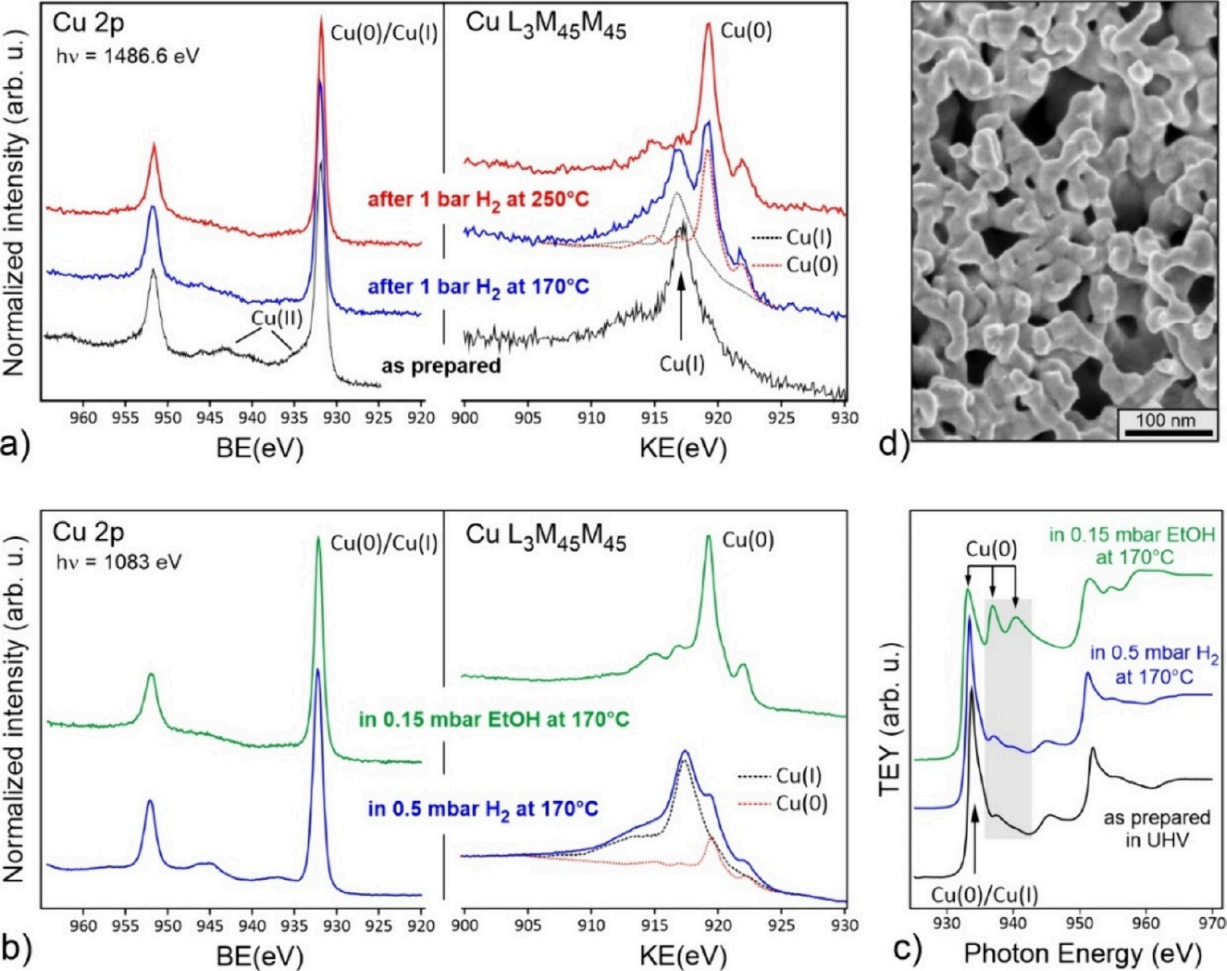
(a) Quasi in situ XPS spectra of Cu_2_O NCs drop-casted onto a SiO_2_/Si­(100) substrate recorded with an Al Kα X-ray source (*h*ν = 1486.6 eV). The Cu 2p and Cu LMM Auger spectra were obtained before (“as prepared”) and after sequential reduction in 1 bar of H_2_ at 170 and 250 °C for 30 min performed in a high-pressure cell. (b,c) Synchrotron NAP-XPS spectra (b) and X-ray absorption near edge structure (XANES) spectra of the Cu L_3_ and Cu L_2_ edges (c) obtained on nc-Cu_2_O in 0.5 mbar H_2_ and then in 0.15 mbar of EtOH, both at 170 °C. XANES spectrum of the “as prepared” sample in UHV is shown for comparison. (d) Typical morphology of the nc-Cu_2_O drop-casted samples after NAP-XPS measurements.

Synchrotron NAP-XPS spectra of Cu_2_O NCs in 0.5 mbar of H_2_ at 170 °C revealed a partially reduced surface (Figure 5b). Not surprisingly, the degree of reduction obtained at low pressures (24%) is much lower than 56% obtained for the sample treated in 1 bar of H_2_ at the same temperature. Remarkably, the Cu(0) concentration, determined from analysis of the corresponding XANES spectrum (Figure 5c) as described in ref 30, is considerably smaller than that derived from the Cu LMM Auger spectrum (Figure 5b), i.e., 10% vs 24%. Such a difference originates from different probing depths of XPS and TEY-XANES spectra which are 1.5 nm^31^ and about 10 nm,^32,33^ respectively. Therefore, the results suggest that metallic Cu formed in the partially reduced catalysts at 170 °C is mostly located at the surface rather than in the bulk. Remarkably, replacing 0.5 mbar of H_2_ with 0.15 mbar of ethanol at 170 °C led to full reduction, which is also observed in the corresponding XANES spectra, suggesting a bulk transformation (Table S2). This finding fully agrees with the above-presented XRD data showing a stronger reductive ability of ethanol compared to hydrogen. On the basis of the NAP Cu 2p and Cu LMM Auger spectra, we can conclude that the Cu surface remains fully metallic under the EDH reaction conditions in the temperature range studied (170–250 °C). Finally, we note that the samples after the NAP-XPS measurements exhibited a sponge-like morphology (Figure 5d), indicating that the particles coalesce even at such low pressures. Obviously, this process intensifies at atmospheric pressures unless the Cu_2_O NCs are mixed with nanosilica, which definitely reduces but cannot fully suppress the particle coalescence (Figure 4).

To shed light on the nature of adsorbed species through analysis of C 1s and O 1s NAP-XPS spectra, we had first to remove the adventitious carbon in the “as prepared” nc-Cu_2_O samples that obscured the observation of ethanol derived species. For this, we exposed the sample to 0.5 mbar O_2_ at 250 °C that, indeed, reduced the C 1s signal to a negligible level (Figure S3a). However, such treatment caused the Cu­(I) → Cu­(II) phase transformation clearly seen in the corresponding Cu 2p, Cu LMM, and XANES (Figure S3) spectra, but the cubic shape of the particles remained unchanged (Figure S4).

The C 1s and O 1s NAP-XPS spectra measured in 0.5 mbar O_2_ at 250 °C are depicted in Figure 6. The remaining C 1s signal at 288.1 eV is tentatively assigned to traces of formate.^34^ The O 1s signal at 529.0 eV originates from the lattice O ions in CuO, whereas the peak at 531.0 eV is usually associated with nonstoichiometric Cu suboxide.^35,36^

**Figure 6:**
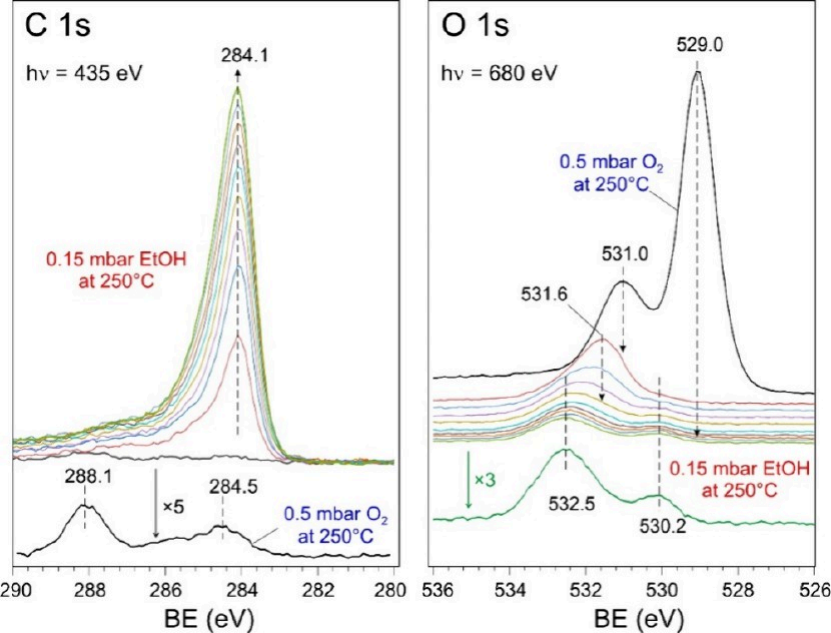
C 1s and O 1s NAP-XPS spectra of Cu_2_O NCs drop-casted on a gold foil. The sample was first exposed to 0.5 mbar O_2_ at 250 °C to remove adventitious carbon. Then oxygen was pumped out without sample cooling, and 0.15 mbar of EtOH was introduced into the chamber. The spectra were continuously recorded, as indicated by dashed arrows. The time interval between the spectra was ca. 1.5 min. The C 1s spectrum measured in O_2_ before ethanol exposure and the last O 1s spectrum measured during the reaction with ethanol are reproduced with the *y*-axis scale enlarged by a factor of 5 and 3, respectively. The spectra are offset for clarity.

Upon exposure to ethanol at 250 °C, the O 1s peaks at 529.0 and 531.0 eV disappear almost immediately (within a few minutes), giving rise to the signal at 531.6 eV. The latter can be assigned to intermediate Cu­(I) species, most likely surface hydroxide, formed during the Cu­(II) reduction to Cu(0); see Figure S3a. The 531.6 eV signal decreases in time and ultimately disappears, while a new signal at 532.5 eV appears together with a weak signal at 530.2 eV. Only these two signals remain at the steady state reaction. The 532.5 eV state is at an energy considerably higher than ever reported for the Cu oxides, and as such it should be attributed to ethanol derived ad-species. (Note, however, that a high BE (533.2 eV) has been recently assigned to interstitial O atoms in the bulk regions of Cu_2_O, only accessible with hard X-ray photoelectron spectroscopy.^37^) The 530.2 eV state is close to that found in initial nc-Cu_2_O (530.5 eV, Figure S3a) and therefore can be assigned to traces of Cu­(I). However, it can also be attributed to O adatoms on fully reduced, metallic Cu.^38^

In the C 1s region, the signal at 284.1 eV appeared immediately upon ethanol exposure. The observed peak is asymmetric, but its intensity scaled only with the reaction time, suggesting that the entire peak belongs to individual species. The binding energy is characteristic for C–C bonds like in carbon, with the long tail at higher BEs indicating C–H species. There is no direct correlation between the intensities of the O 1s at 532.5 eV, which stays almost constant, and the C 1s at 284.1 eV, which continuously grows up with time. Note also a much higher concentration of the respective C containing species compared to the O related counterpart. All of these findings favor the assignment of the C 1s signal to O-deficient carbon deposits like coke. The O 1s peak at 532.5 eV peak is, in fact, close to molecular adsorbed ethanol, which is characterized by O 1s at 532.9 eV and C 1s at 285.7 eV, respectively.^39^ Apparently, the respective C 1s signal from ethanol is screened by the signal from the coke. Importantly, the ethoxy species were not detected under these reaction conditions, as they should manifest themselves by O 1s at 531.0 eV and C 1s at 285.5 eV, respectively.^39^ This finding suggests fast transformation of ethoxy into acetaldehyde that desorbs immediately upon formation and also various CH_*x*_ fragments, ultimately resulting in coke.

Finally, we note that, due to the severe surface charging of the nc-Cu_2_O/SiO_2_ samples and strong contribution of C and O signals from the silica “support”, the XPS analysis could only be performed for the state of Cu, which showed virtually identical spectral evolution as for unsupported Cu_2_O NCs discussed above. In particular, Cu 2p, Cu LMM, and XANES spectra did not reveal any sign of oxidized Cu species in an ethanol atmosphere (not shown here).

### 3.3 Diffuse Reflectance Infrared Fourier Transform Spectroscopy (DRIFTS)

To gain more insight into the state of the catalyst during the reaction, we carried out DRIFTS measurements. First, we address the results obtained for pure nc-Cu_2_O. Figure 7a displays consecutive DRIFTS spectra recorded on the “as prepared” sample in the H_2_/Ar flow during heating from room temperature to 250 °C and then at constant temperature 250 °C for 1 h. The strongest band at 652 cm^–1^ falls in the range of the Cu­(I)–O bond stretching frequencies reported in the literature for Cu_2_O nanoparticles and films, although the reported values considerably depend on the synthesis.^40–42^ It appears that a sharp band at 794 cm^–1^ also belong to the Cu–O phonon bands, whereas other spectral features are associated with various adsorbates formed upon washing the synthesized Cu_2_O nanocubes in EtOH/H_2_O and subsequent drying in air. A broad band centered at ∼3300 cm^–1^ and a peak at 3604 cm^–1^ correspond to the ν­(OH) stretching vibrations in H-bonded and isolated OH species, respectively, and the band at 1610 cm^–1^ corresponds to the δ­(OH) bending mode. Multiple bands in the 2950–2850 cm^–1^ region (C–H stretch), and the peaks at 1096, 1052 (C–O stretch), and 896 cm^–1^ (C–C stretch) can be assigned to both ethoxy and traces of molecularly adsorbed ethanol, since their respective bands overlap.^43–46^ The 1600–1350 cm^–1^ region shows several bands which can be associated with adventitious CO_2_ adsorption. Upon heating in H_2_/Ar, the ν­(Cu–O) band at 652 cm^–1^ loses intensity and ultimately disappears at 250 °C; meanwhile ad-species either desorb (the bands at 2957, 1096, 1052, and 896 cm^–1^ disappear) or transform into other species such as acetates (1567–1540 and 1440 cm^–1^).^47,48^ Finally, all bands disappear, indicating the formation of clean metallic Cu, although some carbonaceous residues may be present at the surface as shown by XPS.

**Figure 7:**
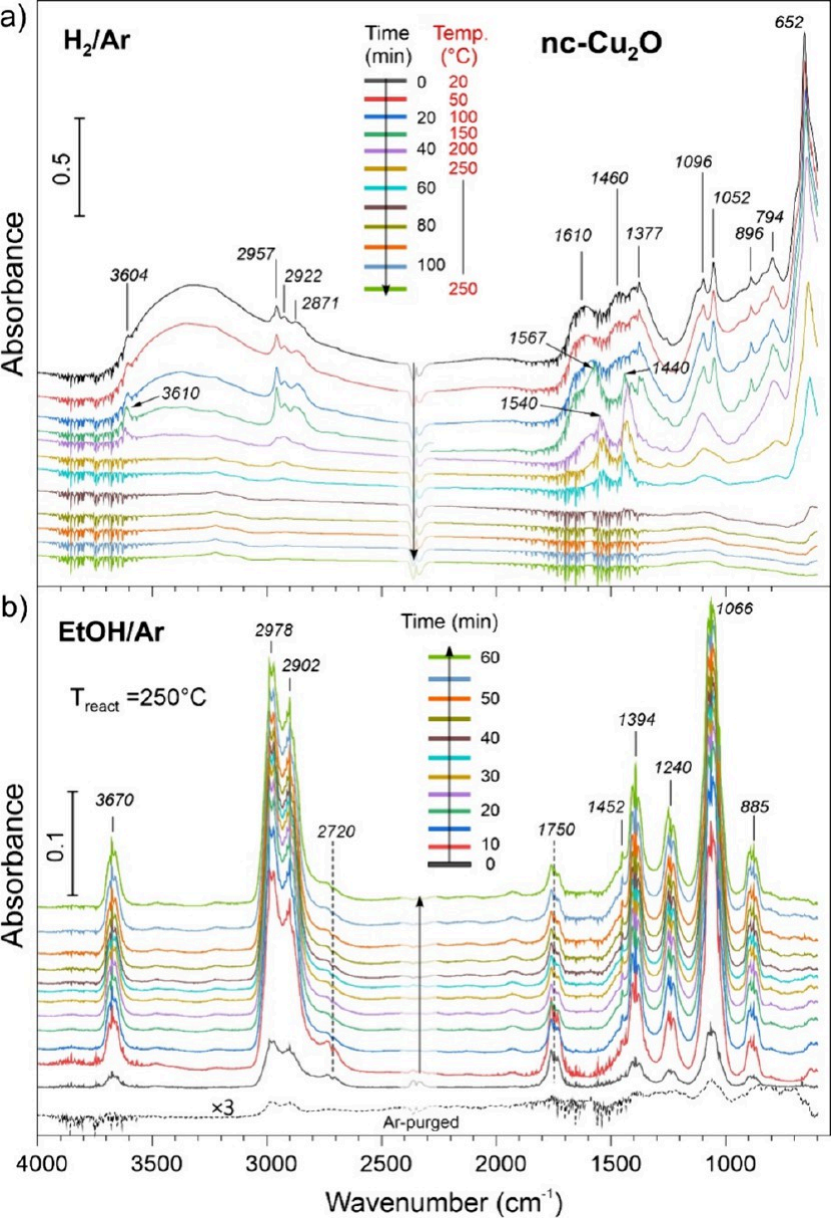
(a) In situ DRIFTS spectra recorded on nc-Cu_2_O in the H_2_/Ar flow during heating from room temperature to 250 °C (at a rate of 5 °C/min) and then at 250 °C for ca. 50 min. Subsequently, the gas flow was switched to EtOH/Ar (b). The consecutive spectra are referenced to the spectrum measured in pure Ar before switching to an EtOH/Ar flow. The dashed line shows the spectrum (enlarged by a factor of 3) after purging the DRIFTS cell with Ar at 250 °C for 30 min. In both panels, the spectra are offset for clarity.

Figure 7b displays in situ DRIFTS spectra measured on the reduced sample in the EtOH/Ar flow at 250 °C. (To better identify reaction induced spectral changes, it is common to use spectra divided to a reference spectrum measured right before the admission of ethanol. The original (unreferenced) spectra are depicted in Figure S5.) The spectra revealed all characteristic bands of ethanol in the gas phase, i.e., 3670 cm^–1^ (OH stretch), 2978 cm^–1^ (CH_3_ asymmetric stretch), 2902 cm^–1^ (CH_2_ asymmetric stretch), 1452 cm^–1^ (CH_3_ asymmetric deformation), 1394 cm^–1^ (CH_3_ symmetric deformation), 1240 cm^–1^ (OH bending), 1066 cm^–1^ (C–O stretching), and 885 cm^–1^ (C–C stretching). The bands at around 1750 cm^–1^ (C=O stretch) and 2720 cm^–1^ (C–H stretch) are fingerprints of acetaldehyde formed during the reaction.^44,46^ After the DRIFTS cell was purged with Ar at 250 °C, only traces of ethanol were detected, most likely trapped in the pores.

DRIFT spectrum of the nc-Cu_2_O/SiO_2_ catalyst represents, in essence, a superposition of individual spectra of nc-Cu_2_O and SiO_2_ (top spectrum in Figure 8a), where the bands at 1987, 1870, 1130, and 810 cm^–1^ belong to silica,^49,50^ and the band at 648 cm^–1^ belongs to the Cu_2_O phase. The ν­(OH) region features isolated silanols (Si–OH) at 3740 cm^–1^ and a broad band at ∼3500 cm^–1^ that comprises H-bonded silanols, surface hydroxyls on Cu_2_O NCs, which all contribute to the δ­(OH) band at 1626 cm^–1^. Heating at 250 °C in the Ar flow resulted in only partial dehydroxylation (Figure S6). In particular, no decomposition of the Cu_2_O phase occurred. Spectral changes during sample heating to 250 °C in the H_2_/Ar flow were found to be virtually identical to those obtained separately on nc-Cu_2_O and SiO_2_. Silica undergoes partial dehydroxylation, while the ν­(Cu–O) band at 648 cm^–1^ fully disappears. Therefore, in full agreement with the XRD, TEM, and XPD results, Cu_2_O NCs transform into the metal Cu particles “supported on” or “diluted by” partially dehydroxylated SiO_2_.

**Figure 8:**
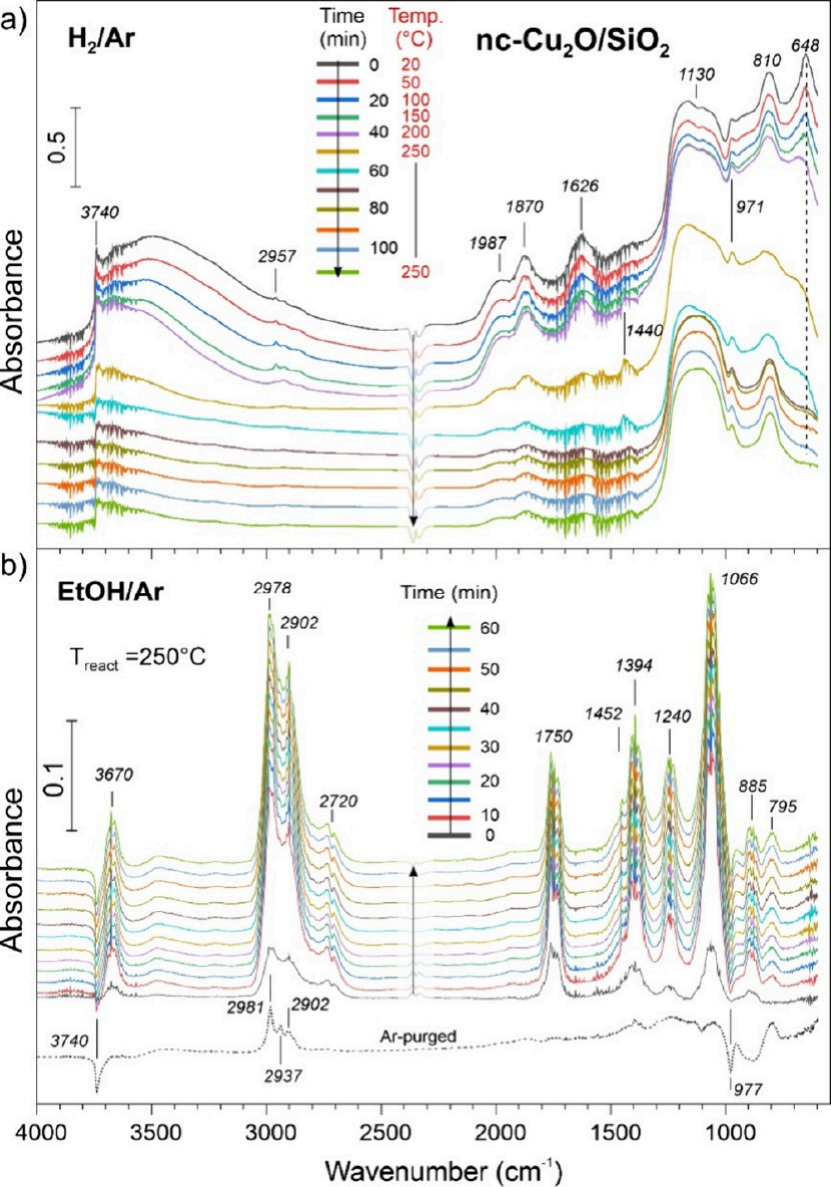
(a) In situ DRIFTS spectra recorded on nc-Cu_2_O/SiO_2_ in the H_2_/Ar flow during sample heating from room temperature to 250 °C (5 °C/min) and then at 250 °C for ca. 50 min. Subsequently, the gas flow was switched to EtOH/Ar (b). The consecutive spectra are referenced to the spectrum measured before the admission of ethanol. The dashed line shows the spectrum after purging the DRIFTS cell with Ar at 250 °C for 30 min. In both panels, the spectra are offset for clarity.

Figure 8b displays in situ DRIFTS spectra measured on the reduced nc-Cu/SiO_2_ catalyst in the EtOH/Ar flow at 250 °C. The results of a similar experiment at 170 °C are shown in Figure S7. In both cases, the spectra are dominated by ethanol and acetaldehyde in the gas phase, with the intensity of acetaldehyde related bands at 250 °C being significantly higher than that at 170 °C, i.e., in full agreement with the reactivity tests. Two sharp “negative” peaks at 3740 and 977 cm^–1^, which correspond to ν­(OH) and ν­(Si–OH) in isolated silanols, develop within the first minutes of reaction. Simultaneously, a new band appears at 795 cm^–1^ that falls in the range of both δ­(Si–O) and ν­(Si–C) frequencies.^51^ These three bands remain after sample purging with Ar at 250 °C, in addition to the ν­(CH_*x*_) bands at 2981, 2937, and 2902 cm^–1^. All of these spectral features were fully reproduced in the blank experiments on pure silica (not shown here). Therefore, we assigned them to the irreversible reaction of ethanol with isolated silanols most likely resulting in stable ethoxy species.^44^

We also monitored the evolution of the nc-Cu_2_O/SiO_2_ catalyst when it was directly exposed to ethanol atmosphere without the prereduction in H_2_ (Figure 9). Again, ethanol adsorption on silica resulted in the disappearance of the bands at 3744 and 976 cm^–1^ and the appearance of the band at 795 cm^–1^ as discussed above. Remarkably, the formation of acetaldehyde (1750 and 2720 cm^–1^) took place in the first minutes of reaction, although no considerable changes were found in the ν­(Cu–O) region, suggesting that the nc-Cu_2_O phase is also active in EDH. However, after 15 min on stream, a negative signal appeared at 648 cm^–1^ indicating the onset of the Cu_2_O reduction. The signal gains in intensity and saturates after ca. 25 min on stream due to complete Cu­(I) reduction, which is accompanied by an increase in the production of acetaldehyde, as evidenced by the increased intensity ratio of acetaldehyde and ethanol bands at 2720 and 2978 cm^–1^, respectively. The results fully support our conclusion that the increase in activity during the initial stage of reaction on the untreated nc-Cu_2_O catalysts (Figure 2) is associated with ethanol-induced reduction of the Cu­(I) phase.

**Figure 9:**
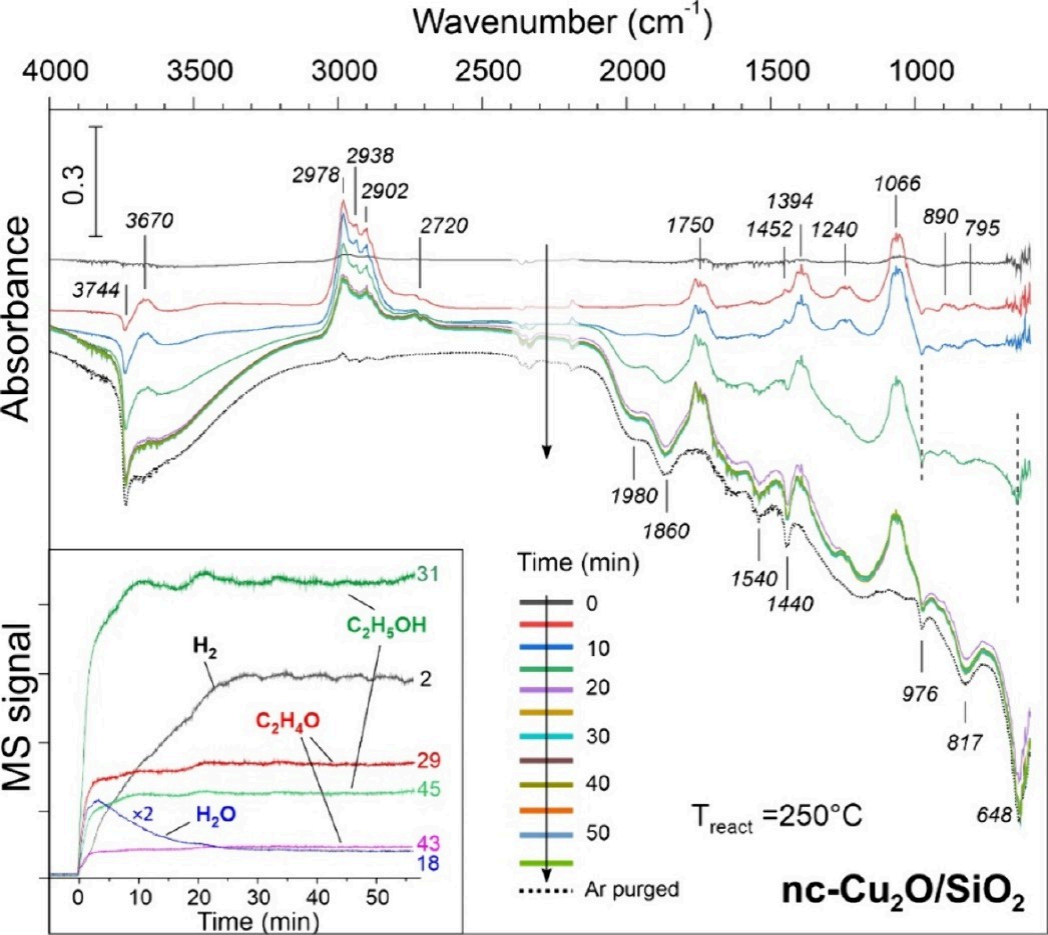
In situ DRIFTS spectra recorded on the “as prepared” (without prereduction) nc-Cu_2_O/SiO_2_ catalyst in the EtOH/Ar flow at 250 °C as a function of time on stream. The dashed line spectrum was obtained after the ethanol flow was stopped, and the cell was purged with Ar at 250 °C for 30 min. The spectra are referenced to the spectrum recorded in pure Ar at 250 °C before admission of ethanol and are offset for clarity. Simultaneously recorded MS signals of hydrogen (2 amu), water (18 amu), ethanol (31, 45 amu), and acetaldehyde (29, 43 amu) are shown in the inset.

This scenario is further substantiated by analysis of the online mass spectra (MS) of the gas phase in the DRIFT cell (inset in Figure 9). The MS data revealed the formation of water before H_2_ evolution sets in. Moreover, we observed the inverse relationship between H_2_O and H_2_ production within the first 20 min of the reaction. Obviously, in the initial stage, ethanol dehydrogenation on the Cu oxide results in surface hydroxyls which desorb as water, thus reducing Cu­(I) to Cu(0). As the reduction proceeds, ethanol dehydrogenation starts to take place on the metallic Cu surface, resulting in H adatoms that recombine and desorb as molecular H_2_, although water remains to be produced as a side product. Nonetheless, the EDH reaction on metallic Cu dominates the steady state.

In order to further investigate the state of the Cu surface, we measured DRIFTS spectra using CO as a probe molecule. We should note, however, that numerous studies of Cu catalysts showed that the ν­(CO) band position depends on several parameters, such as CO pressure and adsorption temperature, but also on precise sample treatments prior to CO exposure.^49,52,53^ Moreover, the bands often overlap, although it is generally accepted that CO adsorption on the Cu­(II) sites manifests itself in the 2150–2200 cm^–1^ region, while the bands in the 2100–2070 cm^–1^ region are characteristic for the metal Cu surfaces depending on the surface orientation.^54,55^ To date, CO DRIFTS spectra for nc-Cu_2_O were only reported by Huang and co-workers, albeit for much larger nanocubes (400–700 nm). The authors observed weak bands at 2101 cm^–1^ at −100 °C^56^ and at 2108 cm^–1^ at 30 °C,^26^ which were assigned to CO adsorption on the defect sites, since the Cu_2_O­(001) facets were theoretically predicted to be O-terminated and hence inert to CO.

Figure 10 compares the CO DRIFTS spectra measured on nc-Cu_2_O/SiO_2_ catalysts at −140 °C in 10 mbar of CO and after CO evacuation. (Full sets of spectra at increasing CO pressure are shown in Figure S8.) It should be noted that the spectra obtained on the “as prepared” catalysts varied from sample to sample, most likely due to adventitious surface species left upon Cu_2_O synthesis. This is illustrated in Figure 10(a,b) that compares two samples purged with an Ar flow at room temperature for 30 min (a) and 10 h (b) before the CO DRIFTS measurements. On both samples, the spectra are characterized by the main peak and a shoulder at high frequency, both growing at increasing CO pressure (Figure S8). The signal at ∼2155 cm^–1^ only appears at high CO pressures and disappears upon evacuation, thus indicating weak CO bonding. Irrespective of the initial state, all samples after in situ reduction in H_2_/Ar at 250 °C showed the main peak at 2100 (±3) cm^–1^ and a shoulder at 2125 (±5) cm^–1^ (Figure 10c).

**Figure 10:**
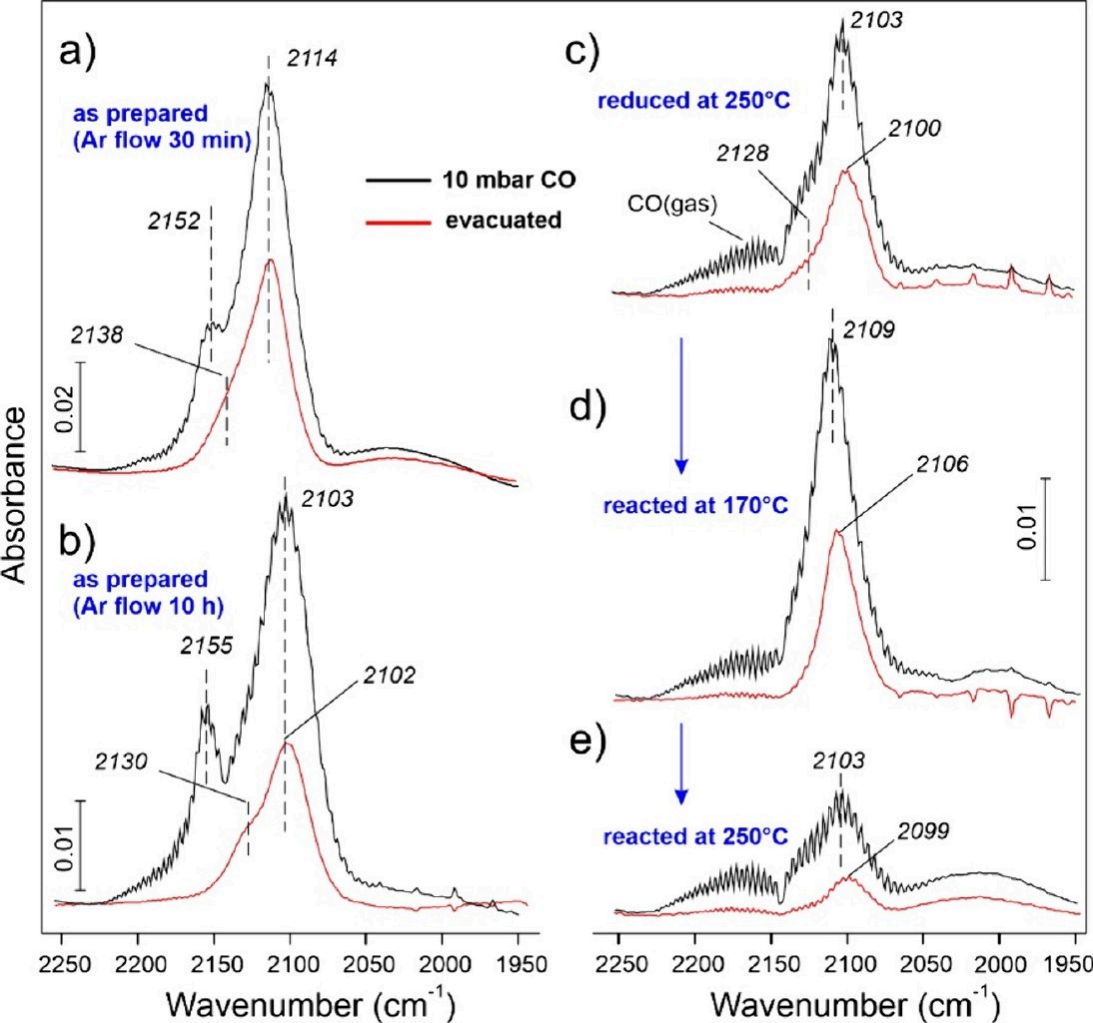
In situ CO DRIFTS spectra recorded at −140 °C in 10 mbar of CO (black lines) and after evacuation (red lines): the “as prepared” nc-Cu_2_O/SiO_2_ sample purged with Ar at room temperature for 30 min (a) and 10 h (b); after subsequent reduction in H_2_/Ar at 250 °C for 2 h (c); then after reaction in EtOH/Ar at 170 °C (d) and at 250 °C (e) for 5 h at each temperature. For panels (c–e), the cell was first purged with Ar at the reaction temperature for 30 min, then pumped out to ∼10^–6^ mbar with sample cooling to −140 °C, and then exposed to CO.

The band at 2155 cm^–1^, often assigned to CO adsorption on silanols,^52,57^ is no longer present after reduction and can therefore be linked to CO adsorption on minority Cu­(II) sites and/or hydroxyls, initially present on the nc-Cu_2_O surface (see Figure 5a, Figure 7a). The spectra obtained for the reduced catalysts featuring the 2100 and 2125 cm^–1^ bands are very similar to those reported for the large (20–60 nm) Cu nanoparticles in the 10 wt % Cu/SiO_2_ catalysts, which have been assigned to CO on Cu(0) and oxidized Cu­(I) sites, respectively,^53^ the latter being attributed to the Cu­(I)–O–Si linkages at the metal/oxide interface. In contrast, Hadjiivanov and Knözinger^49^ assigned the high frequency band (in this case at 2130 cm^–1^) to CO adsorption on Cu(0) sites in 1 wt % Cu/SiO_2_ catalysts.

After the EDH reaction at 170 °C for 5 h performed in the DRIFTS cell, the main band considerably shifts to a higher frequency (i.e., from 2100 to 2106 cm^–1^) without losing intensity. However, the band shifts back to 2099 cm^–1^ after subsequent reaction at 250 °C for 5 h (Figure 10e), but now loses intensity, suggesting substantial decrease of the Cu surface area. The ν­(CO) frequencies higher than 2100 cm^–1^ on the Cu catalysts are usually explained in terms of either incomplete Cu­(I,II) reduction and/or surface roughness (small size) of metallic Cu particles.^52^ Since ethanol behaves as a strong reducing agent, it seems unlikely that an extended Cu oxide layer will form in an ethanol atmosphere, as shown above by NAP-XPS (Figure 6). Even though the formation of a well-ordered “monolayer” Cu_2_O oxide film on the Cu(111) surface was shown to shift the ν­(CO) band from 2077 to 2100 cm^–1^,^58^ such a film was found unstable in the ethanol atmosphere.^59^ On the other hand, the appearance of bands at 2102–2112 cm^–1^ on the single crystal Cu(001) surface was explained by the CO induced formation of Cu adatoms and small Cu clusters.^55^ Therefore, we can tentatively conclude that the EDH reaction at 170 °C is accompanied by the formation of a highly corrugated Cu surface manifested via the 2106 cm^–1^ band, whereas the Cu surface appears to be well-ordered at high reaction temperature (250 °C) due to the enhanced Cu adatoms mobility, and thus showing the same band at 2100 cm^–1^ as for the reduced catalyst, although a substantial part of the Cu surface is blocked for CO adsorption by carbon species, as observed in the NAP-XPS experiments.

### 3.4 Catalyst Stability. Comparison with “Conventional” Cu Catalysts

The results of short-term catalytic tests, shown in Figure 2, already revealed a slight decrease in the ethanol conversion within the first hours of reaction, most notably at 230 °C. This is further illustrated in Figure 11 that compares acetaldehyde and hydrogen production kinetics on the untreated and prereduced nc-Cu_2_O/SiO_2_ catalysts during the 12 h of time on stream (TOS). While the reaction at 170 °C shows, in essence, no deactivation (even after 100 h of TOS, see Figure S9), the activity at 250 °C drops by about 50% of the original value within the first 5 h. The deactivation at 250 °C considerably depends on the catalyst prereduction, with a relatively better stability being observed for the “as prepared” catalyst.

**Figure 11:**
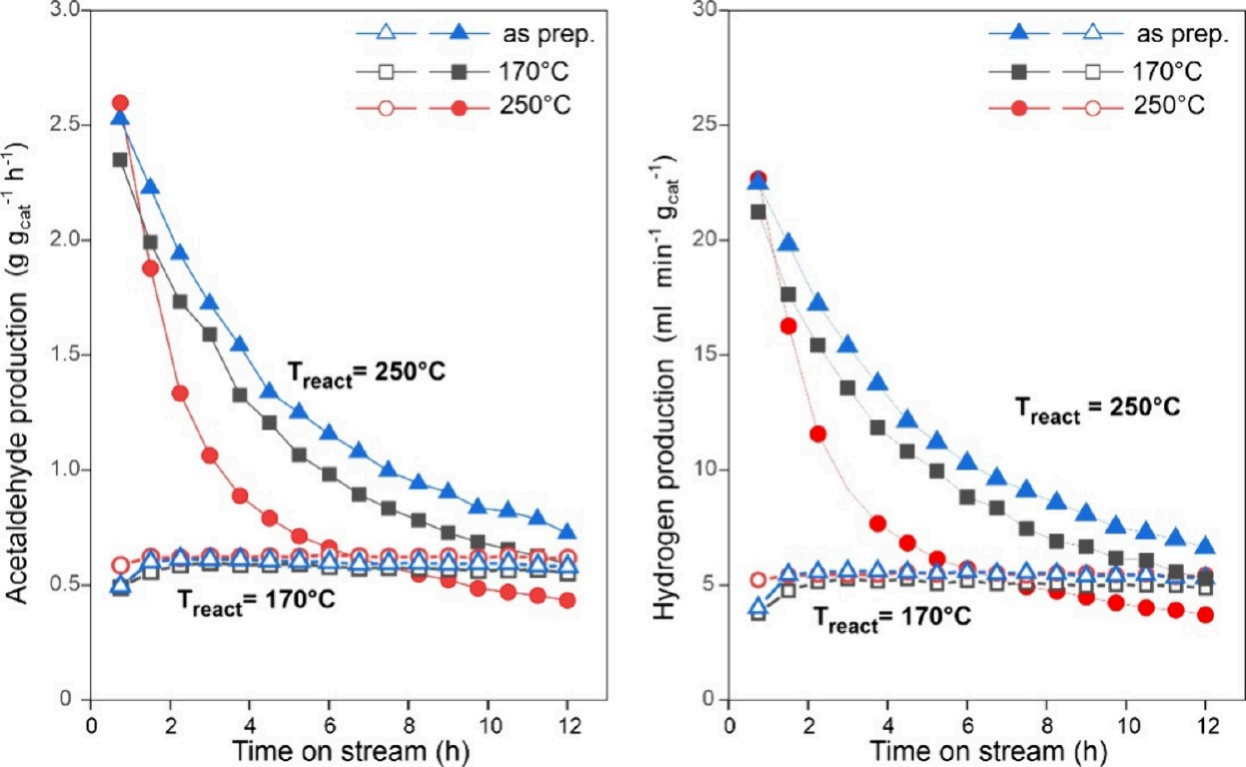
Reactivity of nc-Cu_2_O/SiO_2_ catalysts in ethanol dehydrogenation at 170 and 250 °C as a function of time on stream. Three catalysts are compared: “as prepared”, and after in situ reduction in H_2_/N_2_ at 170 and 250 °C, respectively.

The observed catalyst deactivation at 250 °C is caused by coke deposition rather than metal sintering. Indeed, the latter should have already occurred during the catalyst reduction either in H_2_ at 250 °C (see Table 1) or within the first hours in ethanol flow over the “as prepared” catalyst. In agreement with the NAP-XPS results (Figure 6), in situ Raman spectra revealed carbon formation (Figure 12a). Two spectral features at 1572 and 1330 cm^–1^ are commonly discussed in terms of G and D bands of carbon, where the G band represents the ideal graphite-type lattice vibrations for sp^2^-coordinated C atoms, and the D band mostly reflects disordered carbons in sp^3^-coordination.^60,61^ Note that the band intensities considerably varied across the sample surface, indicating the carbon deposits to be not uniformly distributed (on the scale of the laser beam spot, i.e., 3–5 μm). Based on the D/G intensity ratio, the graphitic component is rather small, because the reaction temperature is too low for graphene layers formation. A relatively large width of the D band also suggests a coexistence of several types of carbon, which are also reflected in the temperature-programmed oxidation profile of the spent catalyst performed in the Raman cell (Figure 12b). Simultaneous desorption peaks of CO_2_ and water observed at ∼80, 320, and 370 °C point to its origin from a thermal decomposition of the same entities, presumably of bicarbonate-type. The broad and featureless CO_2_ signal between 200 and 600 °C can be assigned to the Cu-assisted combustion of coke, with the metallic Cu surface dissociating O_2_ that reacts with carbon species.

**Figure 12:**
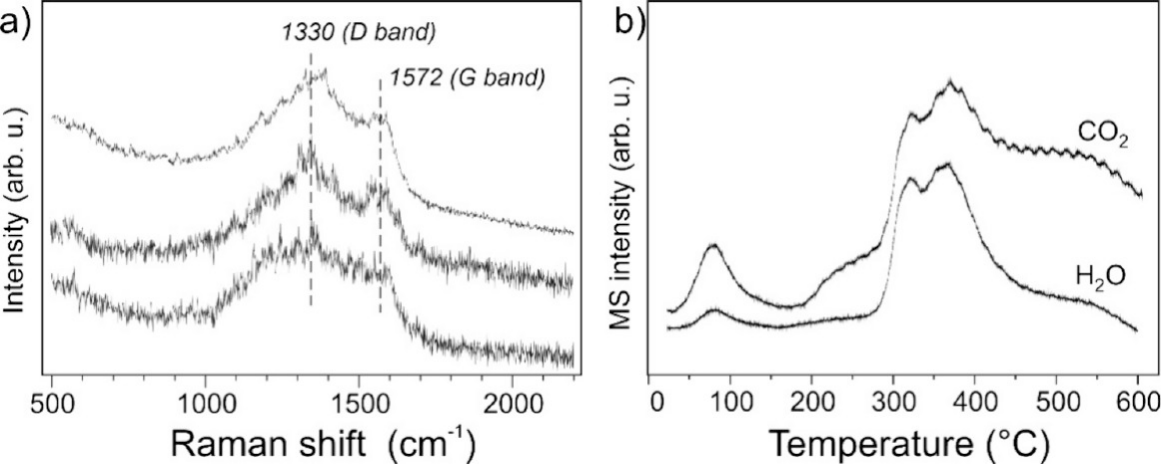
(a) Raman spectra (at 785 nm) obtained on the “as prepared” nc-Cu_2_O/SiO_2_ catalyst after 12 h of the EDH reaction at 250 °C performed in the Raman cell. The spectra, normalized to the peak intensity at 1572 cm^–1^, were recorded at room temperature in the N_2_ flow at several different spots. (b) CO_2_ (44 amu) and water (18 amu) mass-spec signals obtained during heating of the spent catalyst in the Raman cell in a 20 vol % O_2_/N_2_ flow with the rate 5 °C/min.

To evaluate the efficiency of the nc-Cu_2_O/SiO_2_ catalysts compared to the “conventional” silica-supported Cu catalysts, we performed the catalytic test for a 30 wt % Cu/SiO_2_ catalyst prepared by impregnation of the same silica support with a Cu­(II) nitrate precursor followed by calcination at 400 °C (see Methods and Materials). After reduction at 250 °C, the catalyst showed Cu nanoparticles with a rather broad particle size distribution (Figure S10a). Although the great majority of the Cu NPs were below 10 nm (∼4.5 nm, on average), particles as large as 50 nm were also observed. The specific Cu surface area measured in this catalyst (0.86 m^2^/g) after reduction was found to be considerably higher than that for the reduced nc-Cu_2_O/SiO_2_ catalyst (0.43 m^2^/g), i.e., in agreement with a smaller Cu particle size in the Cu/SiO_2_ catalyst, on average. Note that TEM inspection of the spent Cu/SiO_2_ catalyst also revealed considerable redispersion of the Cu phase (Figure S10b). The spent catalyst showed rather a bimodal size distribution of the Cu particles, with maxima at about 2.5 and 20 nm, which is indicative of Cu metal sintering via the Ostwald ripening mechanism.

Figure 13 compares the catalytic performances of the two catalysts, both of which were reduced in situ at 250 °C prior to the reactivity test. Obviously, the nc-Cu_2_O/SiO_2_ catalyst demonstrates a higher ethanol conversion and acetaldehyde (and hydrogen) production than Cu/SiO_2_. The difference becomes even larger when the production rates are normalized to the Cu surface area in the reduced catalysts. Indeed, the turnover frequency (TOF) for nc-Cu_2_O/SiO_2_ catalyst at 230 °C is about three times higher than for the impregnated Cu/SiO_2_ catalyst, i.e., 1.38 s^–1^ and 0.45 s^–1^, respectively (Figure 13, Table S3). Moreover, the apparent activation energy measured for the nc-Cu_2_O based catalyst is considerably lower than for Cu/SiO_2_, i.e., 44.5 and 54 kJ/mol, respectively (Figure S11). The latter finding suggests that ethanol dehydrogenation on Cu is, in fact, structure sensitive, even though the active phase seems to be metallic Cu in all Cu-based catalysts, at least in the direct EDH reaction studied. The relatively broad size distribution of Cu NPs in our highly loaded catalysts makes the particle size relationship to activity quite uncertain. Nonetheless, it appears that large Cu particles dominating Cu_2_O-based catalysts seem to favor the reaction. In this respect, Cu_2_O nanocubes behave as an efficient and well-defined precursor for the synthesis of highly loaded Cu catalysts.

**Figure 13:**
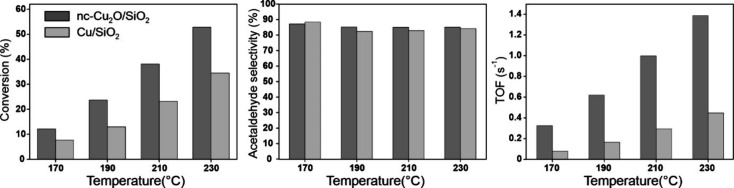
Direct comparison of the catalytic performance of 30 wt % nc-Cu_2_O/SiO_2_ catalyst, prepared by physical mixing, and 30 wt % Cu/SiO_2_ catalyst prepared by Cu­(II) impregnation and subsequent calcination in synthetic air at 400 °C. Both catalysts were reduced in situ in H_2_/Ar at 250 °C prior to the reactivity test.

Finally, comparison of our catalyst with several benchmark Cu/SiO_2_ catalysts reported in the literature (Table 2) also revealed the nc-Cu_2_O based catalyst as a superior catalyst in low-temperature ethanol dehydrogenation.

**Table 2:** Comparison of Catalytic Performances of Silica-Supported Cu Catalysts Reported in the Literature and This Work^a^

Catalyst	*T* (°C)	Reaction conditions	Activation energy (kJ/mol)	TOF^b^ (s^–1^)	Reference
Cu/SiO_2_ (UC)^a^	225–275	Weight/Flow rate = 9.4 min, in 10 mL/min He	54.4	1.7	^62^
Cu/SiO_2_ (FB)^a^			77.8	0.4	
Cu/SiO_2_ (NR)^a^			70.9	0.2	
Cu/SiO_2_	150–200	5.6% EtOH in 10 mL/min He	70		^63^
Cu (SSM)^a^			73		
nc-Cu_2_O/SiO_2_	170–230	0.5 g_EtOH_/h 200 mL/min N_2_	44.5	1.4	This work
Cu/SiO_2_			54	0.45	

a The experimental conditions are indicated.

b Abbreviations: UC, urchin-like, FB, fiber-like, NR, nanorods; SSM, prepared by scarified support method.

c Measured at 225 °C.

## 4 Summary and Conclusions

In this work, we investigated reactivity of Cu_2_O nanocubes (of ∼40 nm in size) in the ethanol dehydrogenation reaction to produce hydrogen and acetaldehyde at moderate temperatures (170–250 °C). The results revealed no substantial differences in the catalytic performance of the catalysts, irrespective of whether they were used “as prepared” or prereduced in H_2_ prior to the reaction. Using microcopy, diffraction, and spectroscopy techniques, we showed that ethanol acts as a strong reducing agent that transforms Cu­(I) oxide into pure metallic Cu in the initial stage of reaction. The reduction process is accompanied by changes of the particle morphology, from a cubic into a roundish shape and substantial Cu sintering. Physical mixing with nanocrystalline silica partially diminishes but cannot fully suppress Cu sintering, which is accompanied by Cu redispersion. The NAP-XPS and DRIFTS results suggest that only the metallic Cu surface is present during the steady state EDH reaction.

Direct comparison with the Cu/SiO_2_ catalyst, prepared by conventional impregnation at the same metal loading, revealed considerably higher activity of the nc-Cu_2_O/SiO_2_ catalyst, if normalized to the Cu content, and even higher activity when normalized to the Cu surface area measured in the reduced catalysts. Although the active phase in this reaction is metallic Cu irrespective of the catalyst’s initial state, the results suggest higher reactivity for larger Cu particles, which are dominated in the catalyst prepared using initially large Cu_2_O nanocubes.

Overall, the results of this study demonstrate that the Cu_2_O nanocubes can be used as a well-defined precursor for synthesis of efficient high loaded Cu catalysts, in particular for monolith type catalysts with a tunable pore structure.

## Supplementary Material



## Notes

Survey XPS spectra of the synthesized Cu_2_O nanocubes, additional NAP XPS spectra in different atmospheres, SEM images of nanocubes, DRIFTS spectra of Cu_2_O-based catalysts at different reaction temperatures, CO DRIFTS spectra of the post-treated samples, and TEM images and catalytic results of “conventional” Cu/SiO_2_ catalysts (PDF)
