# Supplementary material for: Structure and Reactivity of Cu2O Nanocubes in Ethanol Dehydrogenation
Source: ACS Catal. 2025 Oct 21;15(21):18333–47. doi: 10.1021/acscatal.5c06573 (PMC12606080; doi:10.1021/acscatal.5c06573)
Supplement: Supplementary file 1 [file cs5c06573_si_001.pdf]

Supporting Information

Structure and Reactivity of Cu_2O Nano-cubes in Ethanol Dehydrogenation

Van-Canh Nguyen, Eduardo Ortega, Daniel Cruz, Jie Zhu, Wiebke Frandsen,

Shamil Shaikhutdinov,* Beatriz Roldan Cuenya

Department of Interface Science, Fritz Haber Institute of the Max Planck Society, Faradayweg 4-6, 14195 Berlin, Germany

*Corresponding author: shaikhutdinov@fhi-berlin.mpg.de

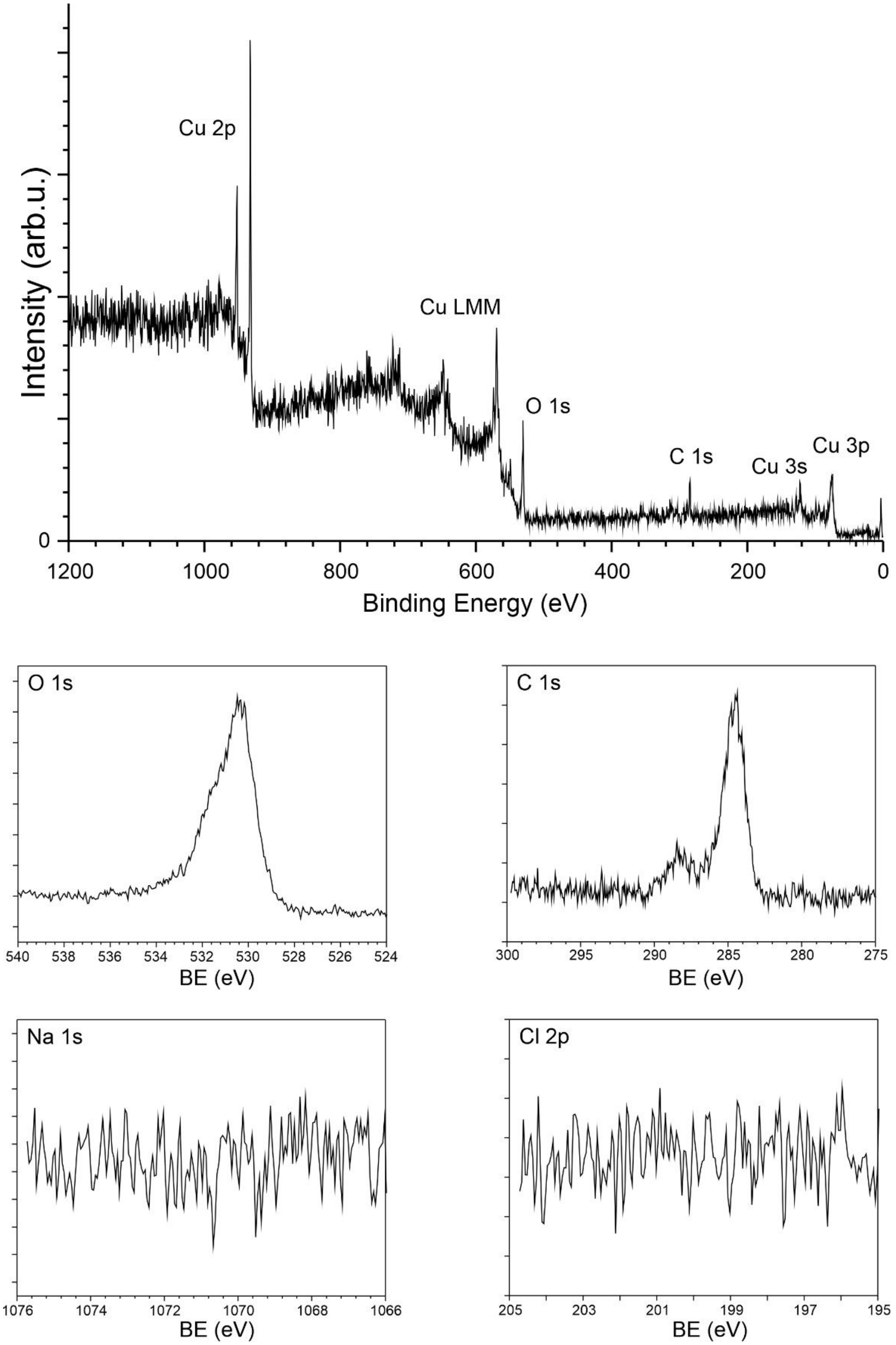

Figure S1. XPS survey spectra of the synthesized Cu_2O nanocubes. The Na 1s and Cl 2p regions are shown to prove the absence of Na and Cl in our samples after washing in ethanol and water and subsequent drying. Adventitious carbonaceous species are detected.

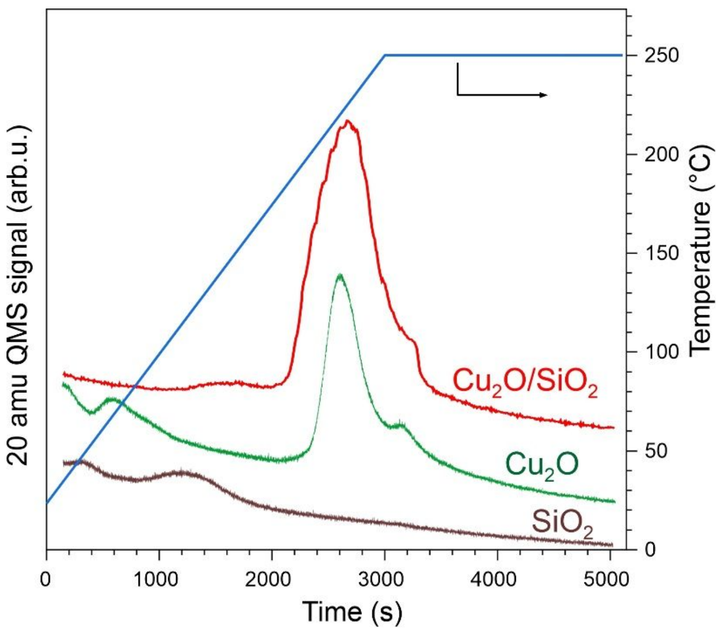

Figure S2. Water formation measured by QMS during heating of pure nc-Cu_2O, pure SiO_2, and 30 wt.% nc-Cu_2O/SiO_2 samples in a 10 vol.% H_2/N_2 flow. The heating rate was 5°C/min. The profiles are offset for clarity.

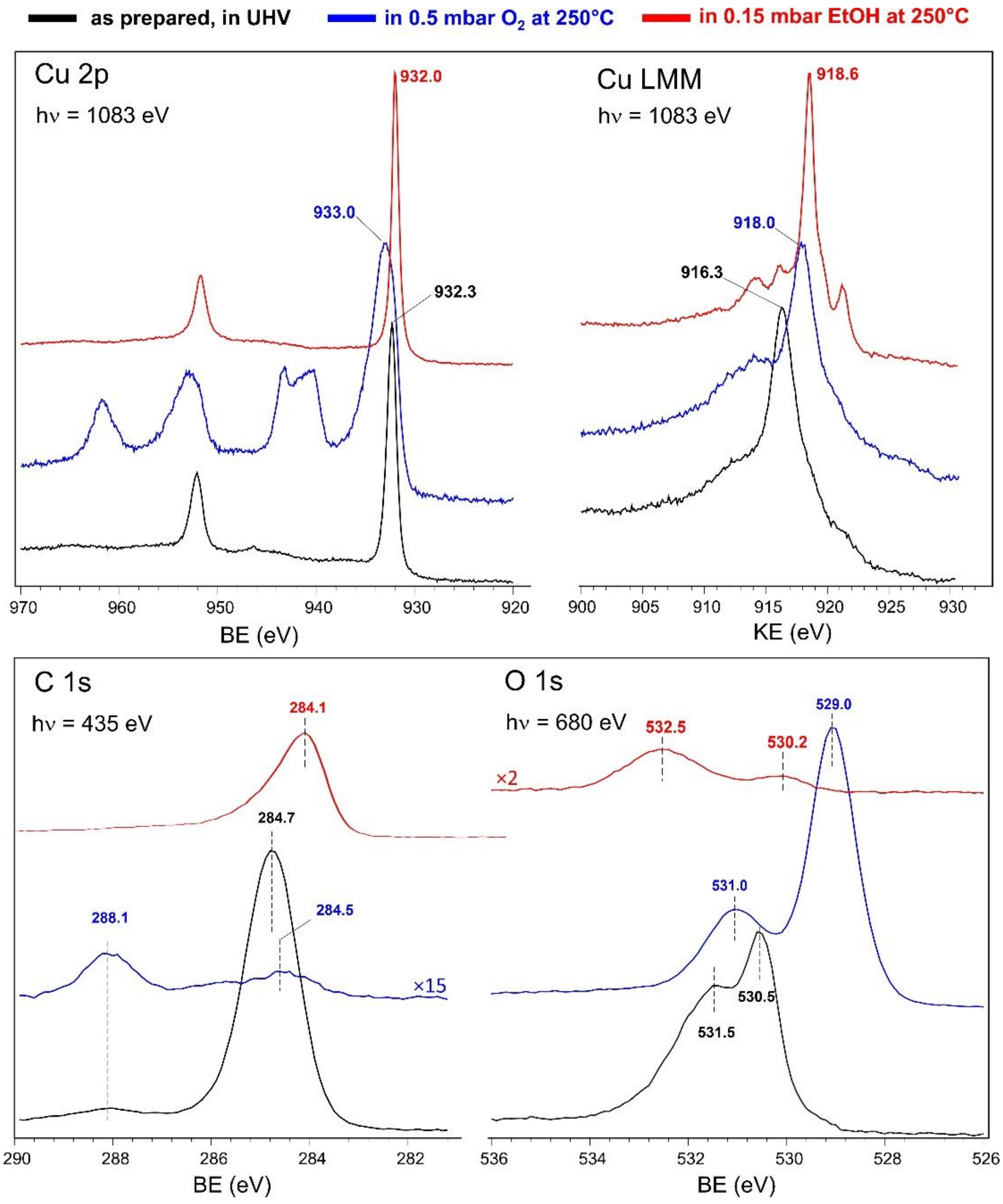

as prepared, in UHV
in 0.5 mbar O2 at 250°C
in 0.15 mbar EtOH at 250°C
Cu 2p
hν = 1083 eV
932.0
933.0
932.3
BE (eV)
Cu LMM
hν = 1083 eV
918.6
918.0
916.3
KE (eV)
C 1s
hν = 435 eV
284.1
284.7
288.1
284.5
×15
BE (eV)
O 1s
hν = 680 eV
532.5
530.2
529.0
×2
531.0
530.5
531.5
BE (eV)

(a)

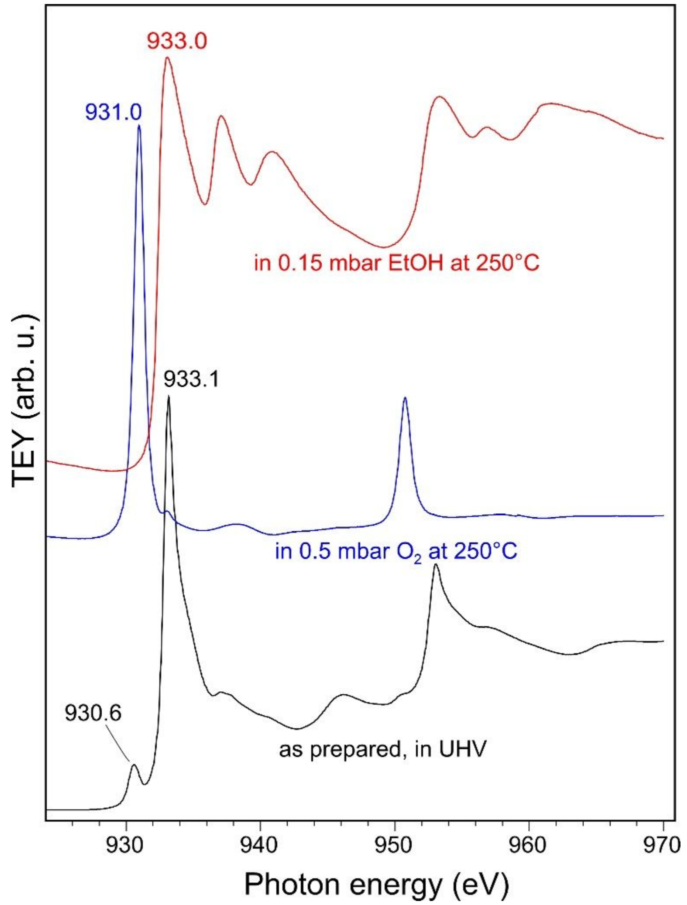

(b)

Figure S3. NAP-XPS spectra **(a)** and Cu L2, L3 edge XANES spectra measured in the total electron yield (TEY) mode **(b)** of Cu_2O nanocubes drop-casted onto a gold foil. The "as prepared" sample was first measured in UHV, then in 0.5 mbar of O_2 at 250°C. Finally, oxygen was pumped out at 250°C and 0.15 mbar of EtOH was introduced. The presented spectra were obtained after ca. 30 min under the conditions as indicated.

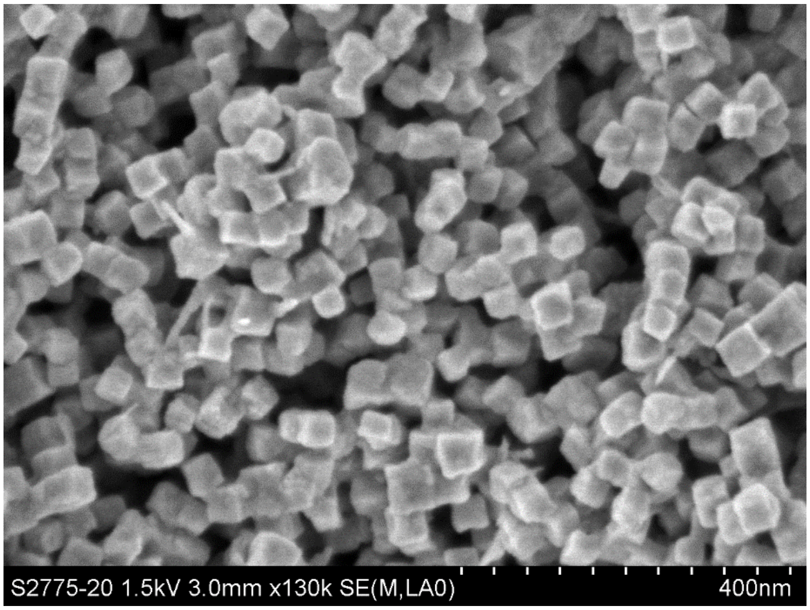

Figure S4. SEM image of Cu_2O nanocubes (drop-casted onto a gold foil) after oxidation in 0.5 mbar of O_2 at 250°C. The cubic shape of the particles is preserved upon the Cu(I) -> Cu(II) oxide transformations observed by XPS and XANES (see Figure S3).

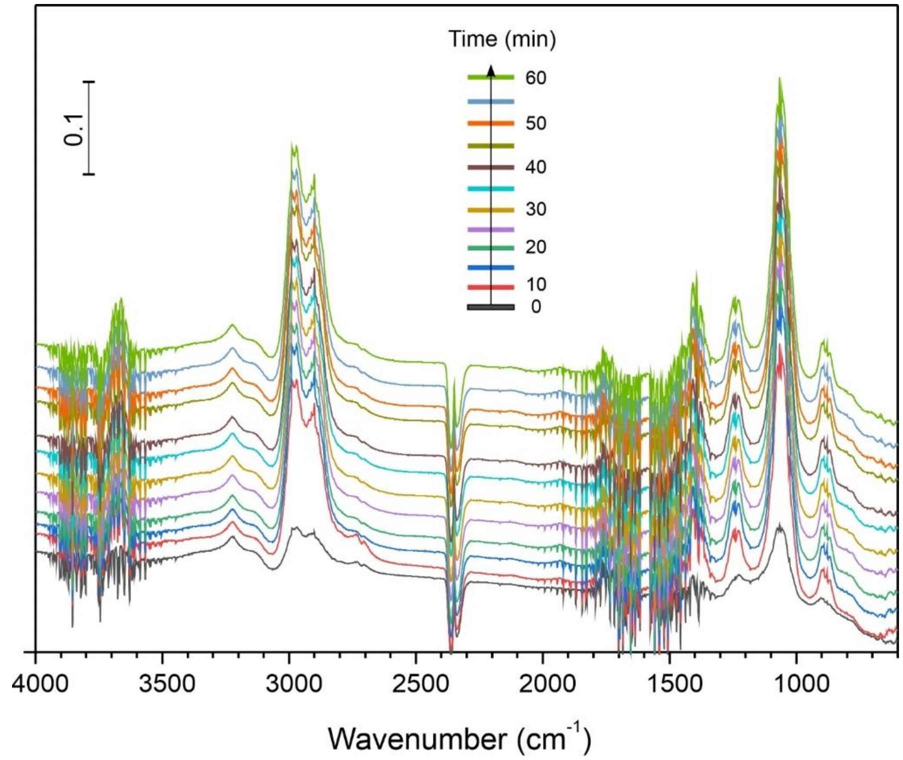

Figure S5. Original (raw) in situ DRIFTS spectra used for Figure 7b, i.e., on unsupported nc-Cu_2O pre-reduced in H_2/Ar at 250°C. The first spectrum (at time zero) was recorded simultaneously with admission of EtOH/Ar at 250°C. The characteristic “noise” is caused by rotational bands of gaseous water molecules present in the optical path of spectrometer. The negative signal at around 2340 cm^{-1} is from the CO_2 gas phase.

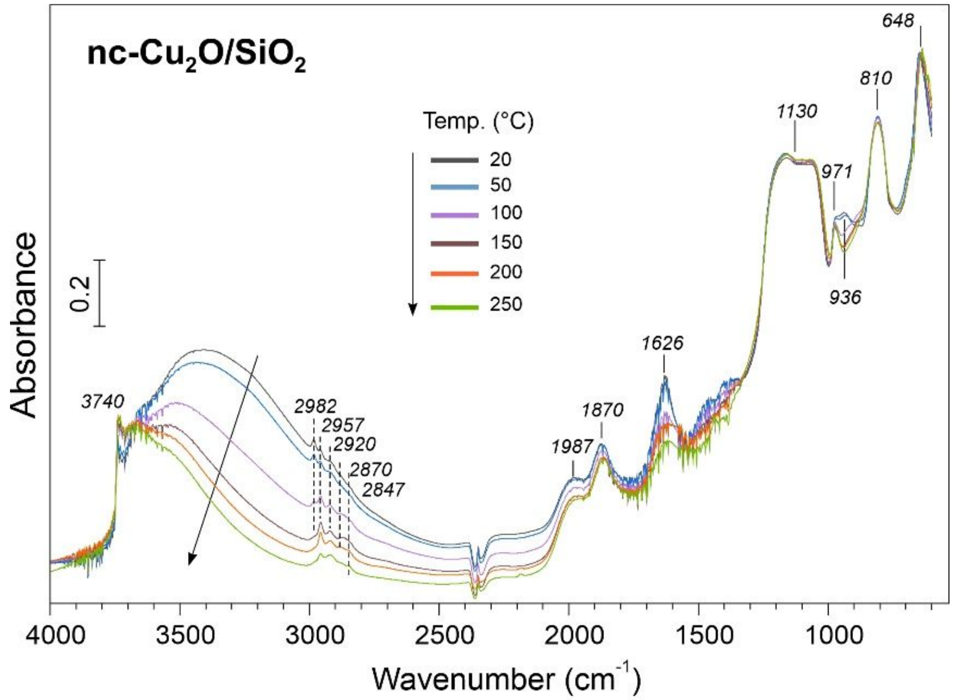

Figure S6. Consecutive DRIFTS spectra recorded during heating the 30 wt.% nc-Cu_2O/SiO_2 catalyst in the Ar flow to the temperatures as indicated. The results show that Cu oxide phase remains (see the band at 648 cm^{-1}) while the silica surface undergoes the dehydroxylation. The bands at 2850 – 2982 cm^{-1} are originated from ethanol/ethoxy species formed during washing/drying of synthesized Cu_2O nanocubes.

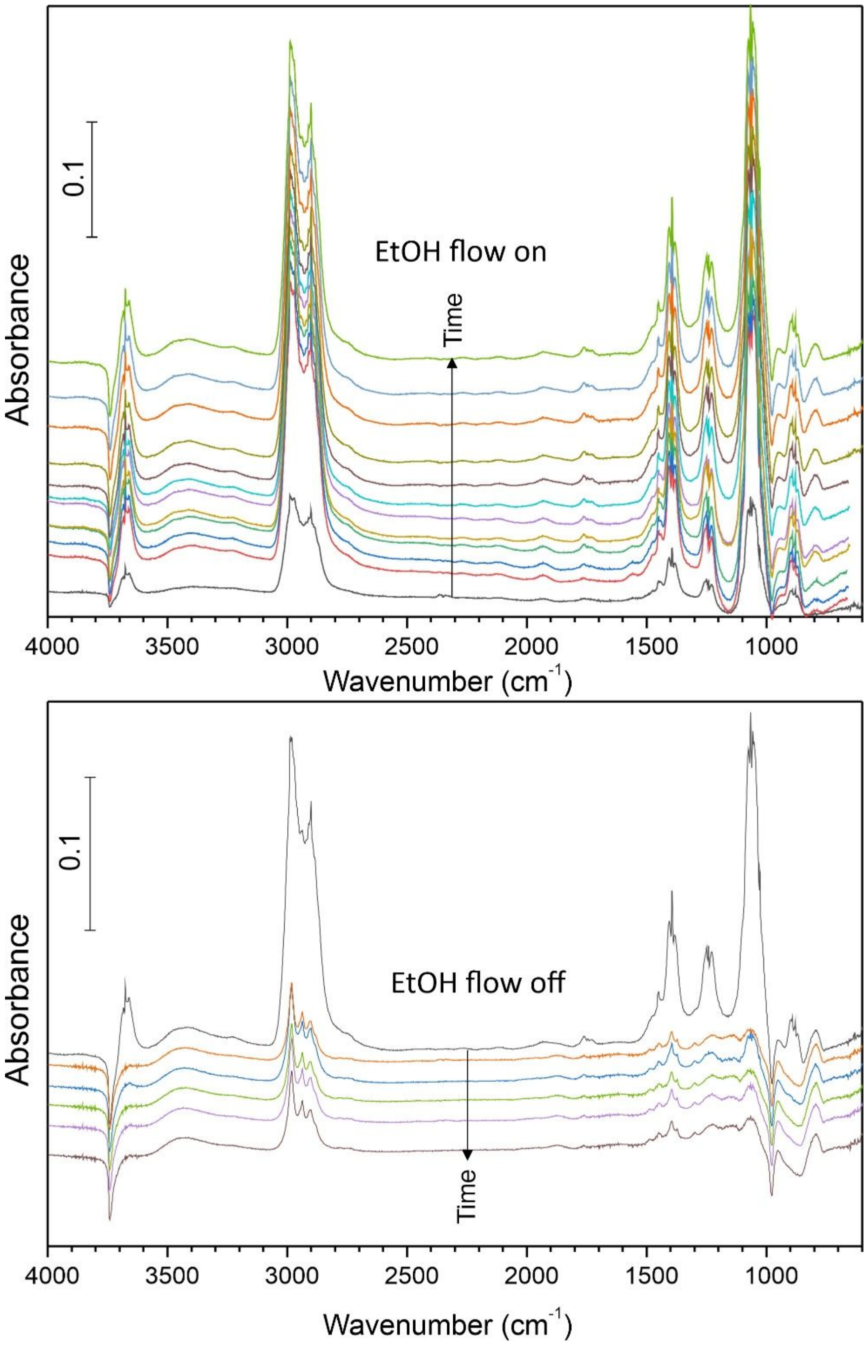

Figure S7. DRIFTS spectra recorded on the nc-Cu_2O/SiO_2 catalyst pre-reduced in situ in 10 vol.% H_2/Ar at 250°C. Top panel: Consecutive spectra (from bottom to top) recorded in the EtOH/Ar flow at 170°C. Bottom panel: Consecutive spectra (from top to bottom) after EtOH flow was stopped. All spectra are referenced to the spectrum measured in the pure Ar flow at 170°C before EtOH was dosed. The acquisition time is 5 min per spectrum.

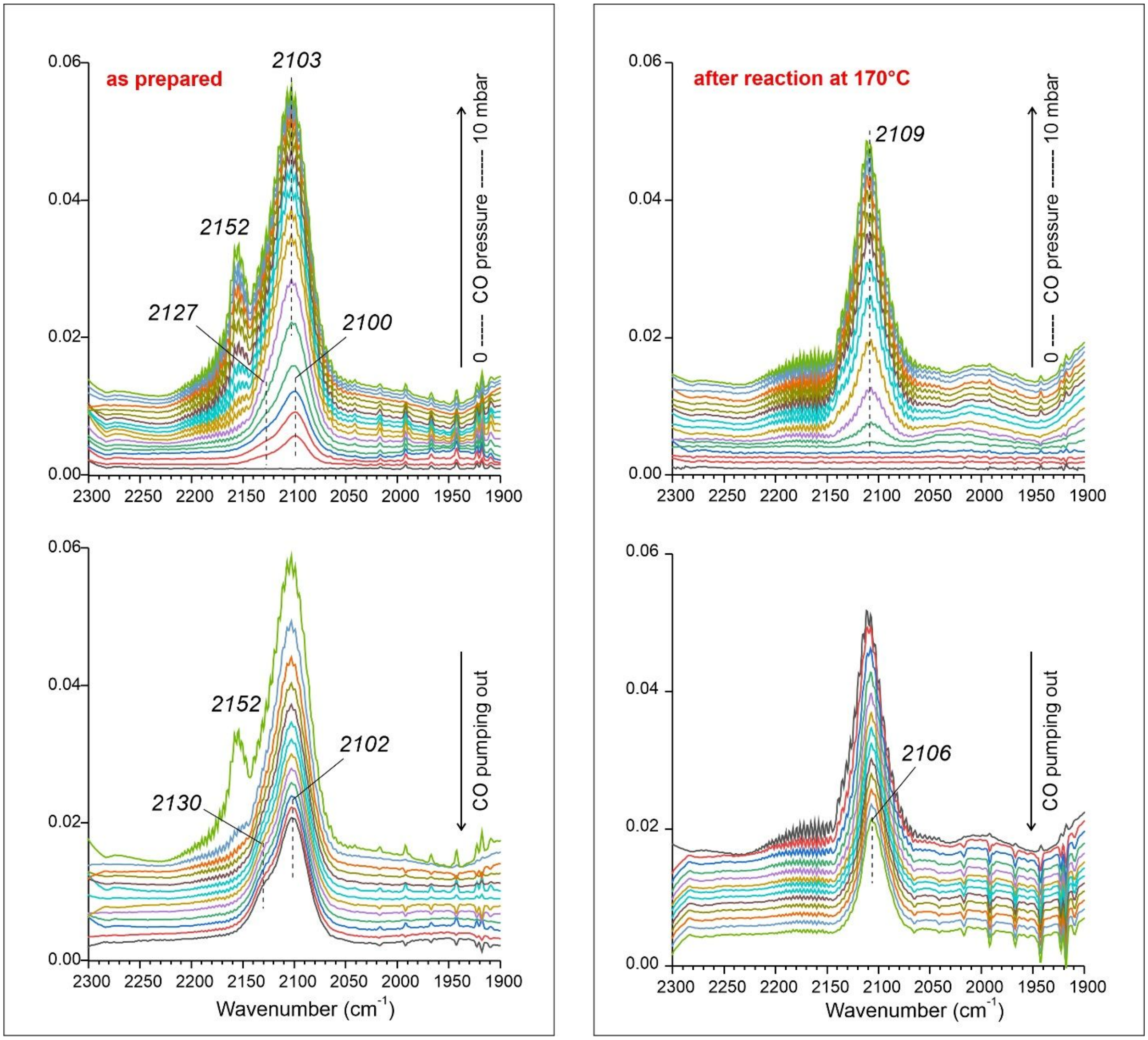

Figure S8. CO DRIFTS spectra obtained on nc-Cu_2O/SiO_2 at -140°C after different treatments as indicated. The spectra were first measured at increasing CO pressure and then after pumping out.

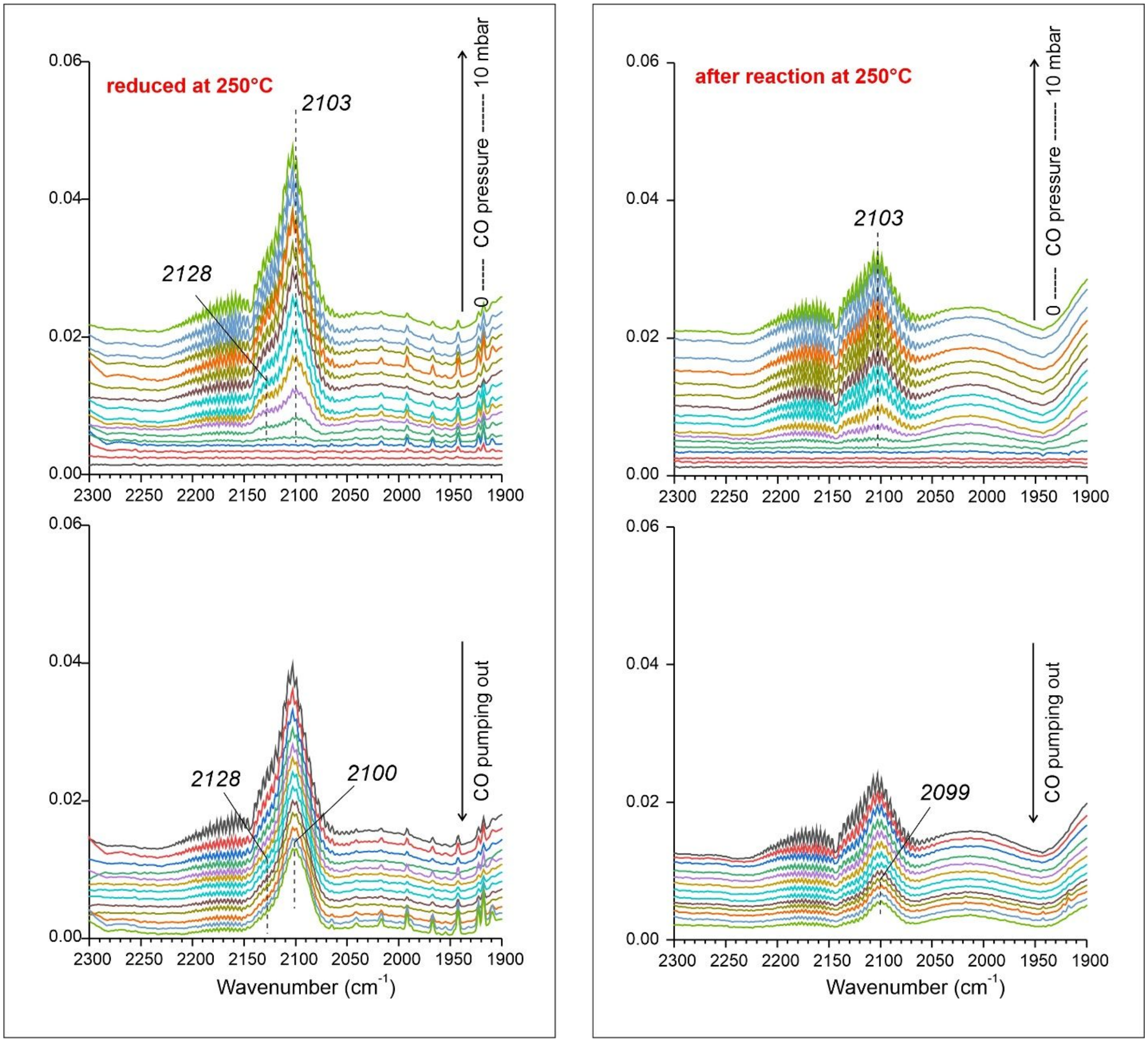

Figure S8 (continued). CO DRIFTS spectra obtained at -140°C on nc-Cu_2O/SiO_2 after different treatments as indicated. The spectra were first measured at increasing CO pressure and then after pumping out.

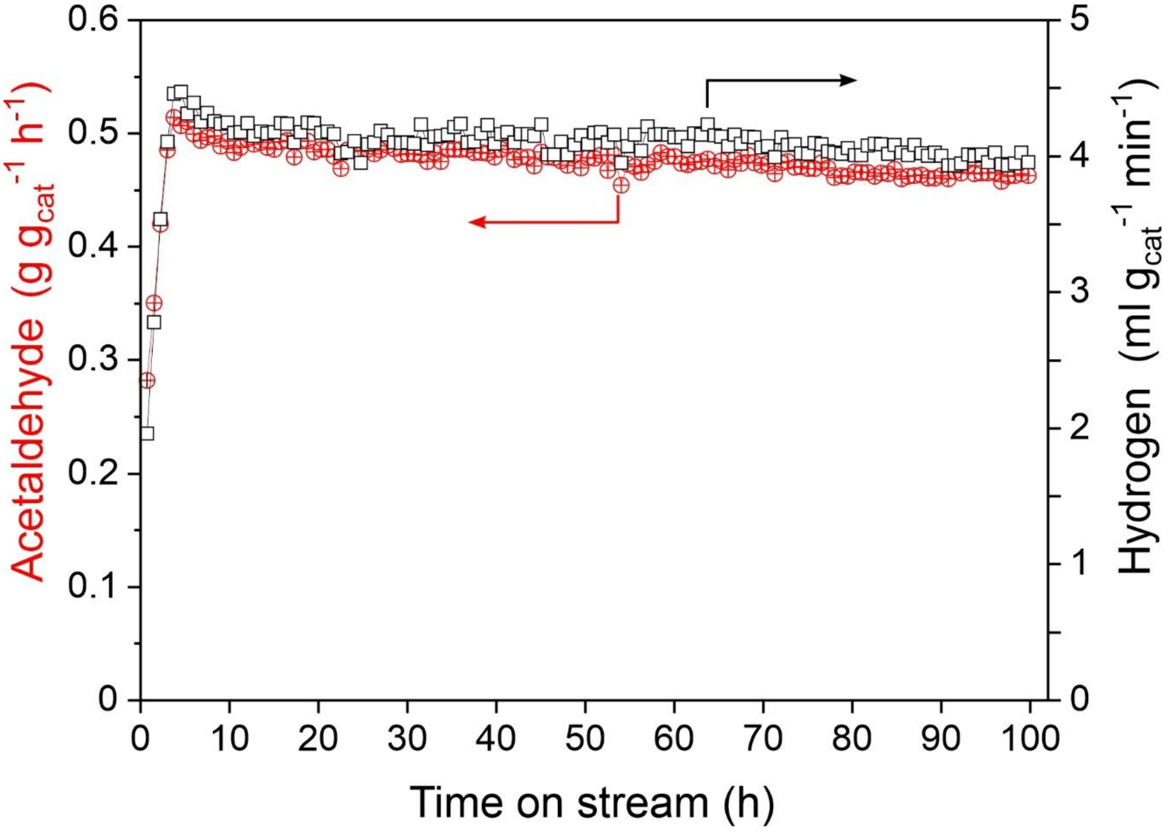

Figure S9. Long-term stability of the nc-Cu_2O/SiO_2 catalyst in ethanol dehydrogenation at 170°C. The catalyst was first heated in N_2 flow (200 ml/min) to 170°C and then mixed with 0.5 g/h of EtOH flow.

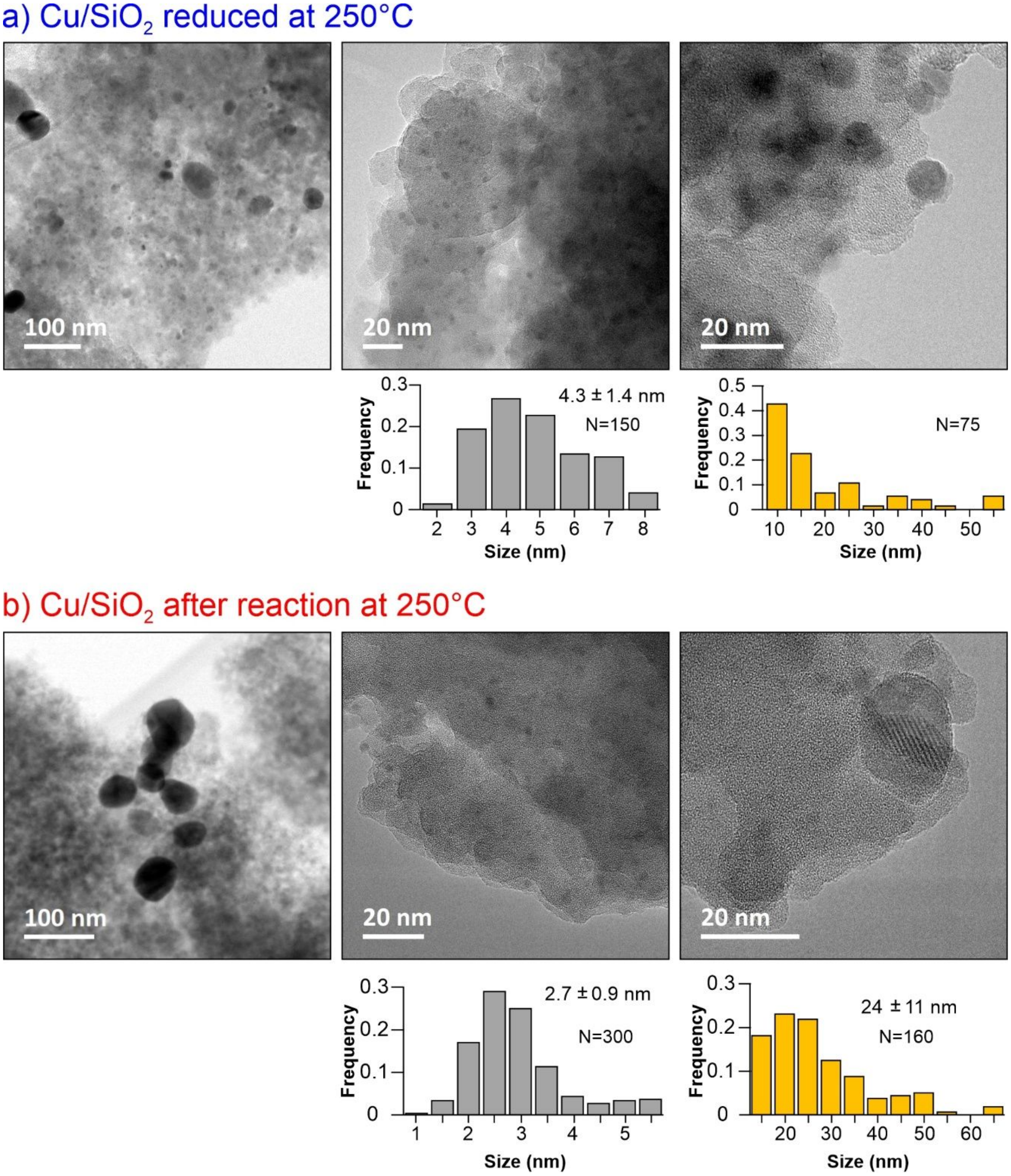

Figure S10. a) Typical TEM images of the "conventional" 30 wt.% Cu/SiO_2 catalyst prepared by impregnation and calcination at 400°C in synthetic air. The catalyst was reduced in 10 vol.% H_2/N_2 at 250°C in the reactor and then transferred to a microscope in the inert atmosphere. The Cu particle size distributions are shown separately for small and large sizes, for clarity. The great majority of the Cu particles is below 10 nm. b) TEM images and size distribution of the same catalyst after 12 h of the EDH reaction at 250°C showing a rather bimodal size distribution with maxima at about 2.5 and 20 nm.

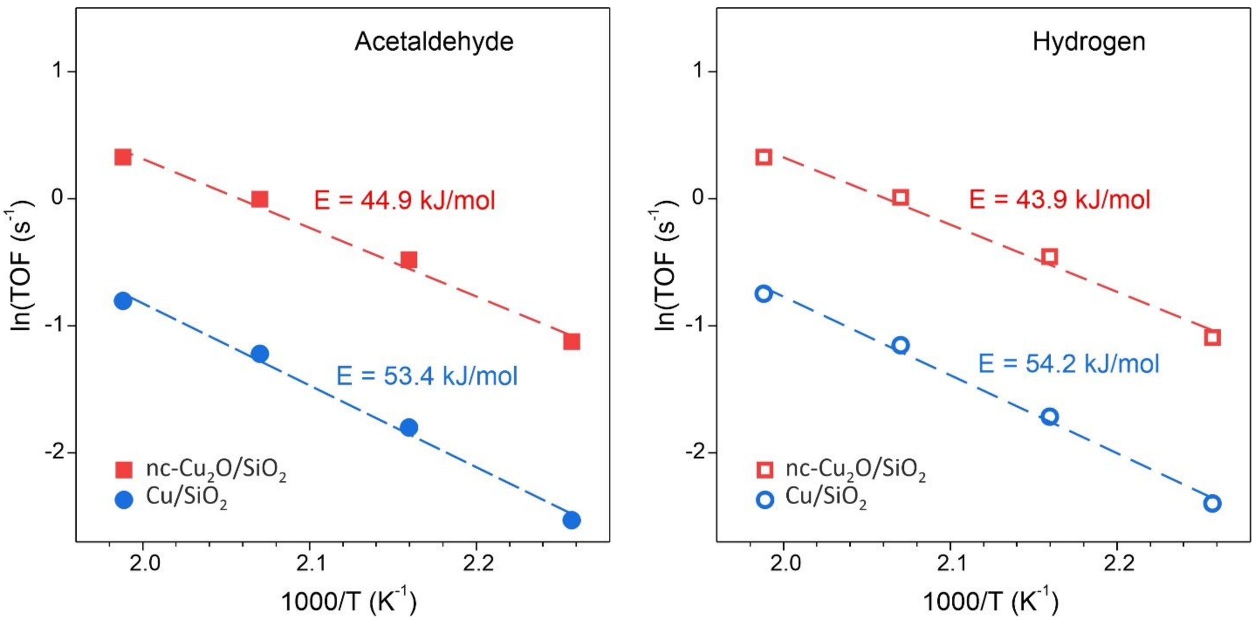

Figure S11. Comparison of Arrhenius plots for the 30 wt.% nc-Cu_2O/SiO_2 catalyst and 30 wt.% Cu/SiO_2 catalyst prepared by conventional impregnation. The catalysts were reduced in situ at 250°C prior to the reaction tests. The results are shown for the production of acetaldehyde (on the left) and hydrogen (on the right).

Table S1. Fraction of the Cu_2O and Cu phases determined by XRD after heating of the 30 wt.% nc-Cu_2O/SiO_2 sample in a 10 vol.% H_2/N_2 flow to the specified temperature increased stepwisely. The corresponding spectra are shown in Figure 3 in the main text.

Temperature (°C)	Fraction (%)	
	**Cu_2O**	**Cu**
170	100	0
190	82	18
210	46	54
230	0	100

Table S2. Fractions of Cu species determined from analysis of XPS and XANES data. Spectra were measured at room temperature in vacuum after certain treatments (quasi in situ XPS) and under reaction conditions (NAP-XPS, XANES) as indicated.

Treatment/Conditions	Cu(0)	Cu(I)
quasi in situ XPS		
after 1 bar H_2 at 170°C	56	44
after 1 bar H_2 at 250°C	100	0
NAP-XPS		
in 0.5 mbar H_2 at 170°C	24	76
in 0.15 mbar EtOH at 250°C	100	0
XANES		
in 0.5 mbar H_2 at 170°C	10	90
in 0.15 mbar EtOH at 250°C	100	0

Table S3. Comparative characteristics of the nc-Cu_2O/SiO_2 catalyst and the "conventional" Cu/SiO_2 catalyst prepared by impregnation. The catalysts were reduced in situ in H_2 at 250°C prior to the catalytic test.

**Catalyst**	**Cu loading**[1] (wt.%)	**Cu surface area in reduced catalysts**[2] (m^2/g_{cat})	**Cu dispersion** (%)	**TOF**[3] (s^{-1})	
				**Acetaldehyde**	**Hydrogen**
nc-Cu_2O/SiO_2	30.3	0.43	0.22	1.39 ± 0.08	1.38 ± 0.07
Cu/SiO_2 (impregnation)	29.8	0.86	0.45	0.45 ± 0.01	0.47 ± 0.01

[1] determined by ICP-MS

[2] measured by N_2O reactive frontal chromatography

[3] measured at 230°C for acetaldehyde and hydrogen
